# Supplementary material for: Allogeneic islet products for type 1 diabetes: Navigating nonclinical and manufacturing regulatory expectations
Source: Stem Cell Reports. 2026 Jul 6;21(7):102998. doi: 10.1016/j.stemcr.2026.102998 (PMC13385429; doi:10.1016/j.stemcr.2026.102998)
Supplement: Document S1. Figure S1 and Tables S1–S6 [file mmc1.pdf]

Stem Cell Reports, Volume *21*

Supplemental Information

Allogeneic islet products for type 1 diabetes: Navigating nonclinical and manufacturing regulatory expectations

Chengyuan Press, Kevin D'Amour, Nicholas Mamrak, David Pepperl, Robert H. Kutner, Diana M. Colleluori, Melanie L. Graham, Michael A. Brehm, Nasir Hussain, Esther Latres, and Marjana Marinac

Figure S1: Example Flow Chart for Product Process Validation

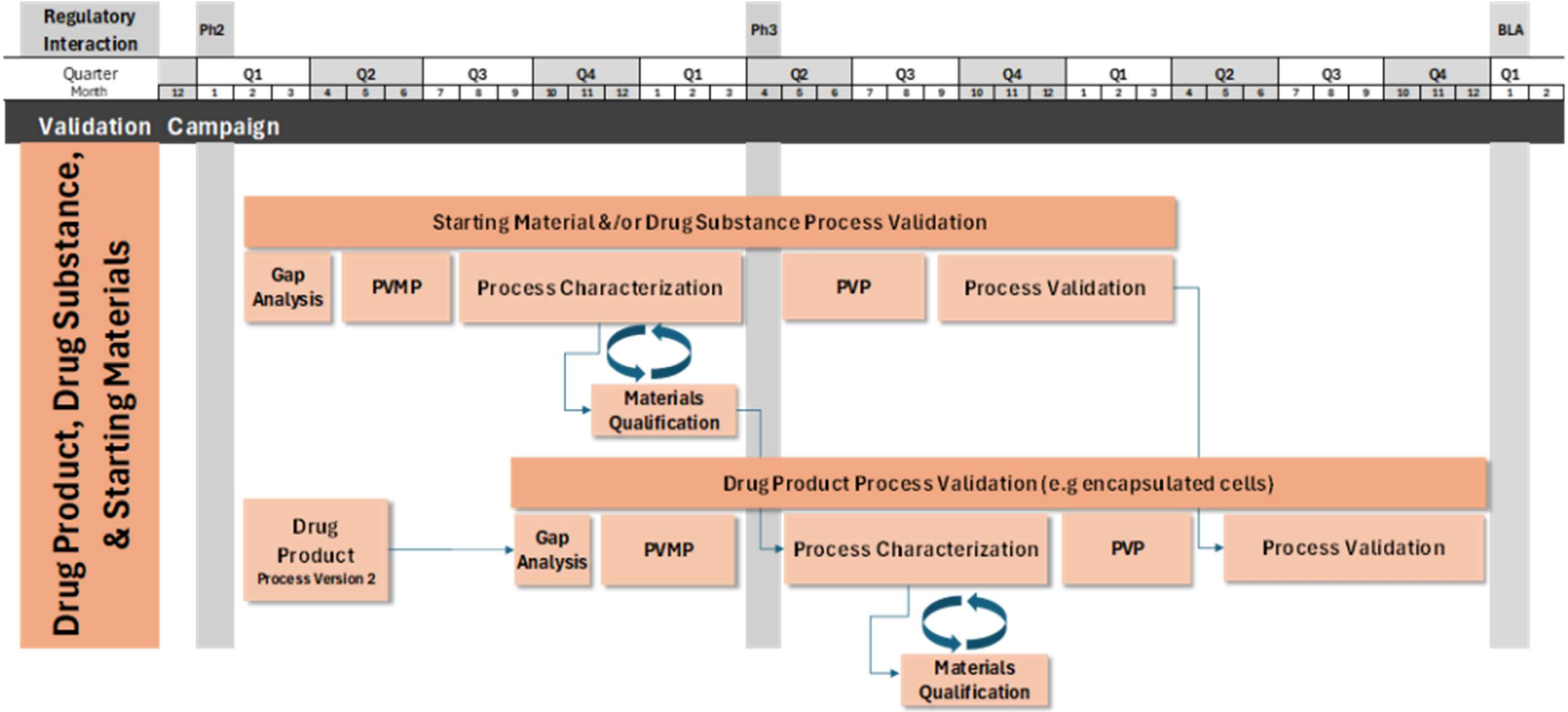

PVP: Process Validation Protocol; PVMP: Process Validation Master Protocol.

Table S1:Nonclinical Regulatory Guidelines for Cellular Therapy Products Across Worldwide Jurisdictions

Region	Regulatory Authority	Product Classification(s)	Regulatory Filing for Clinical Trial	Relevant Regional Guidance**
United States	FDA	CTs<br>GTs<br>TEPs	IND (Investigational New Drug Application) (USFDA, 1995)	• Preclinical Assessment of Investigational Cellular and Gene Therapy Products (USFDA, 2013)<br>o Outlines comprehensive preclinical assessment, focusing on product characterization, mechanism of action, proof-of-concept, biodistribution, toxicology, and dosage for clinical trials<br>• Frequently Asked Questions — Developing Potential Cellular and Gene Therapy Products (USFDA, 2024)<br>o Provides targeted answers for developing CGT products; Considerations for interacting with the FDA (including IND submissions), CMC, nonclinical studies (animal models/toxicology), and clinical trial design/safety
European Union	EMA	ATMP:<br>• GTMPs<br>• SCTMPs<br>• TEMPs	CTA (Clinical Trial Application)	• Guideline on quality, nonclinical and clinical requirements for investigational advanced therapy medicinal products in clinical trials (2024) (EMA, 2025)<br>o Quality control, safety studies, minimally required nonclinical data, and clinical trial design via risk-based approaches for early and late-phase trials
Japan	PMDA	RPs	CTA (Clinical Trial Application)	• Technical Guidance for Quality, Nonclinical Safety Studies and Clinical Studies of Regenerative Medical Products (Human Cell-Processed Products) (2016) (PMDA, 2016)<br>o Essential standards for quality control, nonclinical safety assessment (including tumorigenicity and biodistribution), and clinical study design for human cell-processed products
Australia	TGA	Biologicals:<br>• Class 1<br>• Class 2<br>• Class 3<br>• Class 4	CTA (Clinical Trial Application) or CTN (Clinical Trial Notification scheme)	• Australian Regulatory Guidelines for Biologicals (ARGB) (2018) (TGA, 2018)<br>o Risk-based classification system, manufacturing requirements, and market authorisation processes
Canada	Health Canada	ATPs	CTA (Clinical Trial Application)	• Safety of Human Cells, Tissues and Organs for Transplantation Regulations (2007) (HealthCanada, 2025)<br>o Establish mandatory safety standards for the processing, storage, distribution, and importation of human cells, tissues, and organs (CTO) to prevent disease transmission<br>o Donor suitability assessment (screening/testing), quality management (personnel, facilities), mandatory registration with Health Canada, and error/adverse reaction reporting

FDA: Food and Drug Administration; EMA: European Medicines Agency; PMDA: Pharmaceutical and Medical Devices Agency; CT: Cell Therapy; GT: Gene Therapy; TEP: Tissue Engineered Product; ATMPs: Advanced Therapy Medicinal Product; GTMP: Gene Therapy Medicinal Product; SCTMP: Somatic cell therapy medicinal product; TEMP: tissue engineered medicinal product; RP: Regenerative Medicine products; ATPs: Advanced Therapeutic Products. TGA: Therapeutic Goods Administration. *Typically, in addition to general safety and biodistribution. * and **are not exhaustive lists but the authors' highlights.

Table S2:Comparison of Scientific Meetings and Submissions with Worldwide Regulatory Authorities

**Jurisdiction**	**Early Regulatory Input**	**Accelerated Pathways and Designations**	**Key Features**
USA (FDA)	INTERACT (very early)<br>Pre-IND (prior to IND)	RMAT (Regenerative Medicine)<br>Breakthrough Therapy<br>Fast Track<br>Accelerated Approval<br>Priority Review	• INTERACT meetings for very early-stage products with challenges<br>• Pre-IND meetings help ensure adequacy of IND filing<br>• No fees or costs to any early-stage FDA meetings.<br>• Numerous opportunities for obtaining input and accelerating development process
EU (EMA)	Scientific Advice (SA) Meeting	PRIME (similar to FDA RMAT)<br>Conditional Marketing Authorization<br>Approval under Exceptional Use Marketing Authorization	• Scientific Advice from individual Nations or from Full EU<br>• National scientific advice can vary between jurisdictions, fees may be required<br>• SA from EU takes longer, but more comprehensive, associated with fees<br>• Additional IMPD filing contains comprehensive CMC/nonclinical data<br>• PRIME comparable to FDA's RMAT for regenerative medicine products
Japan (PMDA)	Pre-submission Consult	SAKIGAKE (like FDA Breakthrough Therapy)	• CTA review comparable to US FDA (30 days)<br>• SAKIGAKE pathways affords additional access to PMDA
Australia (TGA)	Pre-submission Meeting	Priority Review<br>Provisional Approval	• Novel, high-risk, FIH therapies such as gene-edited or encapsulated stem cell-derived islets would require CTA unless the FIH trial has been approved by a comparable regulator, then the CTN pathway may be used<br>• CTA involves TGA review of nonclinical and CMC data<br>• GMP compliance for FIH trials is exempted but is required beyond FIH trials, applicable to both CTN and CTA pathways
Canada (Health Canada)	Pre-CTA	Priority Review	• CTA Process highly similar to US IND filing<br>• Priority review speeds review of marketing application<br>• No fees or costs to any early-stage FDA meetings

IMPD: Investigational Medicinal Product Dossier; INTERACT: **I**nitial **T**argeted **E**ngagement for **R**egulatory **A**dvice on **C**BER Produc**t**s

Table S3: Potential Impact of Process Residuals

**Cell Product and Process-Related Impurities**		
**Process-related impurity**	**Method for Measurement**	**Potential Toxicity**
Feeder cells	qPCR using amplicons specific to feeder cell genome sequences	Immunogenicity
Recombinant or animal-derived proteins	ELISA ideally specific to protein	Immunogenicity
Small molecule reagents	HPLC or GC-MS	Direct toxicity
Synthetic mRNA/pDNA	qRT/PCR using amplicons specific for starting material sequences	Genotoxicity, Immunogenicity
Transfection reagents	HPLC differentiating peak-area response times	Direct toxicity
Sorting or purification reagents (e.g., antibodies or beads)	Imaging or secondary sorting measures	Immunogenicity
**Product-related impurities**		
Undifferentiated or incompletely differentiated cells	Flow cytometry for proliferation markers and/or other cell specific markers	Tumorigenicity
Dead cells	Trypan-blue exclusion	Immunogenicity

Table S4: Sample acceptance criteria for MCB and WCB Testing

Test	Acceptance Criteria	
	**Master Cell Bank**	**Working Cell Bank**
Identity	Conforms to specific cell species and donor<br><br>STR profiling	Conforms to specific cell species and donor<br><br>STR profile matches MCB
Cell Growth	Report %viability and total cells	Report %viability and total cells
Potency	Confirmed ability to form cell type of interest	Confirmed ability to form cell type of interest
Genetic Stability	Normal karyotyping<br><br>Molecular testing performed without variants of concern (e.g., NGS or SNP array)	Karyotyping consistent with MCB banding<br><br>Consistent with MCB results
Sterility	No growth	No growth
Mycoplasma	None detected	None detected
Adventitious Agents (*in vivo*)	No adventitious agents detected	N/A
Adventitious Agents (*in vitro*)	No adventitious agents detected	No adventitious agents detected
Viral Contaminants	Free of viral contaminants	N/A
Reprogramming Contaminants*	Clearance of plasmid or viral components (<1 copy per 100 cells)	N/A

STR: Short Tandem Repeat. N/A: Not Applicable, as tests are not generally required for the WCB; *: iPSC-specific consideration (Sullivan et al., 2018)

Table S5: Material Qualification and Testing Requirements

Category	Example(s)	Incoming QC Testing Requirements[1]	Material Qualification Requirements[2]	Re-Qualification[3]
GMP Critical	Primary container/closure	Identify / Appearance<br>Dimension	Full CoA testing of 3 lots, e.g., dimensions, material construction	Annual – Full CoA testing of 1 lot
	Compendial, final formulation, excipient, transport	Appearance	Full CoA testing of 3 lots	Annual – Full CoA testing of 1 lot
	Non-compendial final formulation, transport	Every lot – Full CoA testing	Full CoA testing of 3 lots	Full CoA testing was performed on each incoming receipt
	Primary printed labels	Confirm readability, lot number, expiry accuracy, artwork	Full CoA testing of 3 lots, e.g., dimensions, adhesion, label integrity / functional rub, artwork	Annual – Full CoA testing of 1 lot
Ancillary Tier 1	Licensed product	Appearance, identity	N/A	N/A
Ancillary Tier 2	Compendial grade	Appearance, identity	Full CoA testing or 3 lots	Annual – Full CoA testing of 1 lot
Ancillary Tier 3	Animal-human derived (where the supplier does perform all applicable virus and/or donor level testing)	Appearance, identity	Full CoA testing or 3 lots	Annual – Full CoA testing of 1 lot
Ancillary Tier 4	Animal-human derived (where the supplier does not perform all applicable virus and/or donor-level testing)	Appearance, identity	Full CoA testing or 3 lots and applicable virus testing per 9CFR113 and/or donor-level testing per regulations	Annual – Full CoA testing of 1 lot and applicable virus testing per 9CFR113 and/or donor-level testing per regulations
Single-Use Disposable	Scalpel, Tubing, Filters	N/A	N/A	N/A
Laboratory Reagents	Media, serum	N/A	N/A	N/A

1. For Phase 1 and 2 only verifying the CoA by a GMP-compliant vendor against Raw Materials Specification is typically acceptable for all categories.
2. For Phase 1 and 2 only the Ancillary Tier 4 requirements are expected to meet compliance, all other categories are N/A. It is recommended that such approach is also supported by an internal safety risk assessment.
3. Required for Phase 3 and beyond.

Table S6: Illustrative Examples for Practical Implementation: Mapping Product Attributes to Development Priorities

Product Attributes	Key Nonclinical Question(s)	Preferred Model(s)	Potential Safety Concern	Potency Strategy	CMC/QC Priority
PSC-Derived β-Cells (unprotected)	• Engraftment*<br>• Glucose-responsive function<br>• Dose*<br>• Off-target cell persistence*	• STZ-immune deficient rodent (cell function, long-term, 6+ months)<br>• Non-diabetic immune-deficient rodent (tumorigenicity/distribution)	• Residual PSCs → teratoma*<br>• Genomic instability*	• GSIS*<br>• Markers (e.g., INS, GCG)*	• Residual PSCs and undifferentiated cells (flow cytometry, qPCR)*<br>• Genetic stability (karyotyping, CNV analysis)*
PSC-Derived Progenitors (e.g., pancreatic endoderm)	• *In vivo* maturation timeline<br>• Fate mapping<br>• Ectopic differentiation	• STZ-immune deficient rodent (cell function, long-term, 6+ months)<br>• Non-diabetic immune-deficient rodent (tumorigenicity/distribution)	• Uncontrolled proliferation<br>• Off-target differentiation	• Functional potency after maturation<br>• Markers (e.g., PDX1+, NKX6.1+)	• *In vitro* differentiation to functional endocrine cells<br>• Markers/correlates of *in vivo* maturation potential
Gene-Edited Immune Evasion (e.g., HLA knockout)	• Protection from allo- & autoimmunity<br>• Off-target editing consequences	• Humanized mouse model (e.g., NSG with human immune system)<br>• *In vitro* cytotoxicity assays	• Off-target genomic edits<br>• Insertional mutagenesis<br>• Unanticipated immunogenicity	• Immune evasion (e.g., resistance to alloreactive T cell killing *in vitro*)	• Editing fidelity (targeted or WGS)<br>• Karyotypic stability post-editing<br>• Off-target analysis (e.g., GUIDE-seq)
Encapsulated (immunoisolating)	• Device integrity<br>• FBR<br>• Insulin diffusion kinetics<br>• Route of administration/implant site	• Allogeneic surrogate in immune-competent animal for FBR (e.g., rat, pig)<br>• Immune-deficient rodent for xenograft function	• Fibrotic overgrowth → graft failure<br>• Device rupture<br>• Leachables	• GSIS (pre- and post-encapsulation)<br>• Device integrity<br>• Diffusion (*in vitro* release assay)	• Encapsulation material characterization (biocompatibility ISO 10993)<br>• E&L (ICH Q3E); sterility

*Applies to all categories listed under "Product Attributes" in the table.

References:

EMA. (2025). *Guideline on quality, non-clinical and clinical requirements for investigational advanced therapy medicinal products in clinical trials.* https://www.ema.europa.eu/en/documents/scientific-guideline/guideline-quality-non-clinical-clinical-requirements-investigational-advanced-therapy-medicinal-products-clinical-trials_en.pdf Retrieved from https://www.ema.europa.eu/en/documents/scientific-guideline/guideline-quality-non-clinical-clinical-requirements-investigational-advanced-therapy-medicinal-products-clinical-trials_en.pdf

HealthCanada. (2025). *Safety of Human Cells, Tissues and Organs for Transplantation Regulations (SOR/2007-118).* Retrieved December 3 from https://laws-lois.justice.gc.ca/eng/regulations/sor-2007-118/index.html#:~:text=Table%20of%20Contents,65%20%2D%20Facilities

PMDA. (2016). *Technical Guidance for Quality, Nonclinical Safety Studies and Clinical Studies of Regenerative Medical Products (Human Cell-Processed Products)*. https://www.pmda.go.jp/files/000273883.pdf Retrieved from https://www.pmda.go.jp/files/000273883.pdf

Sullivan, S., Stacey, G. N., Akazawa, C., Aoyama, N., Baptista, R., Bedford, P., Bennaceur Griscelli, A., Chandra, A., Elwood, N., Girard, M., Kawamata, S., Hanatani, T., Latsis, T., Lin, S., Ludwig, T. E., Malygina, T., Mack, A., Mountford, J. C., Noggle, S.,…Song, J. (2018). Quality control guidelines for clinical-grade human induced pluripotent stem cell lines. *Regen Med*, *13*(7), 859–866. https://doi.org/10.2217/rme-2018-0095

TGA. (2018). *Classifying biologicals*. Retrieved December 3 from https://www.tga.gov.au/resources/guidance/classifying-biologicals

USFDA. (1995). *Guidance for Industry: Content and Format of Investigational New Drug Applications (INDs) for Phase 1 Studies of Drugs, Including Well-Characterized, Therapeutic, Biotechnology-derived Products.* https://www.fda.gov/regulatory-information/search-fda-guidance-documents/content-and-format-investigational-new-drug-applications-inds-phase-1-studies-drugs-including-well Retrieved from https://www.fda.gov/regulatory-information/search-fda-guidance-documents/content-and-format-investigational-new-drug-applications-inds-phase-1-studies-drugs-including-well

USFDA. (2013). *Guidance for Industry: Preclinical Assessment of Investigational Cellular and Gene Therapy Products.* https://www.fda.gov/regulatory-information/search-fda-guidance-documents/preclinical-assessment-investigational-cellular-and-gene-therapy-products Retrieved from https://www.fda.gov/regulatory-information/search-fda-guidance-documents/preclinical-assessment-investigational-cellular-and-gene-therapy-products

USFDA. (2024). *GUIDANCE DOCUMENT: Frequently Asked Questions — Developing Potential Cellular and Gene Therapy Products.* https://www.fda.gov/regulatory-information/search-fda-guidance-documents/frequently-asked-questions-developing-potential-cellular-and-gene-therapy-products Retrieved from https://www.fda.gov/regulatory-information/search-fda-guidance-documents/frequently-asked-questions-developing-potential-cellular-and-gene-therapy-products
