# Supplementary material for: Allogeneic islet products for type 1 diabetes: Navigating nonclinical and manufacturing regulatory expectations
Source: Stem Cell Reports. 2026 Jul 6;21(7):102998. doi: 10.1016/j.stemcr.2026.102998 (PMC13385429; doi:10.1016/j.stemcr.2026.102998)
Supplement: Document S2. Article plus supplemental information [file mmc2.pdf]

STEM CELL REPORTS
Review

OPEN ACCESS

Allogeneic islet products for type 1 diabetes: Navigating nonclinical and manufacturing regulatory expectations

Chengyuan Press,[1] Kevin D'Amour,[2] Nicholas Mamrak,[1] David Pepperl,[3] Robert H. Kutner,[3] Diana M. Colleluori,[3] Melanie L. Graham,[4] Michael A. Brehm,[5] Nasir Hussain,[1] Esther Latres,[1] and Marjana Marinac[1,*]

[1]Breakthrough T1D, New York, NY, USA
[2]Independent Consultant, Redwood City, CA, USA
[3]Biologics Consulting Group, Inc., Alexandria, VA, USA
[4]Preclinical Research Center, Department of Surgery, University of Minnesota, Minneapolis, MN 55455, USA
[5]Program in Molecular Medicine, Diabetes Center of Excellence, University of Massachusetts Chan Medical School, Worcester, MA 01605, USA
*Correspondence: mmarinac@breakthrought1d.org
https://doi.org/10.1016/j.stemcr.2026.102998

SUMMARY

Next-generation allogeneic islet cell products—including stem cell (SC)-derived, gene-edited, and encapsulated islets—offer scalable and curative solutions to restore physiological glucose control in type 1 diabetes. The critical path to success for these complex therapies hinges on robust nonclinical pharmacology and toxicology evidence and rigorous, scalable, and well-controlled manufacturing. We discuss strategies for essential nonclinical data for First-in-Human trials, emphasizing SC-specific risks, question-specific model selection, stage-by-stage process characterization, and safety, identity, purity, and potency testing. This paper serves as a resource for developers, highlighting best practices and regulatory expectations to advance transformative T1D cell therapies from bench to clinic.

INTRODUCTION

In people with type 1 diabetes (T1D), glucose levels cannot be properly maintained as the pancreatic islet β-cells are attacked and destroyed by the immune system. This results in dangerously high blood glucose concentrations, leading to multiple short- and long-term severe and life-threatening complications (Kerper et al., 2022). While exogenous insulin therapy has been a life-saving treatment for a century, it cannot replicate the body's natural, precise control of blood glucose by pancreatic islet β-cells. People with T1D on insulin therapy still have a much higher likelihood of experiencing microvascular and macrovascular diseases and a substantial daily burden (Galloway and Chance, 1994; Nathan and Group, 2014).

A new line of research has aimed at developing islet cell replacement therapies that can restore the body's physiological glucose control (Grattoni et al., 2025). Currently, deceased donor islet transplantation is an available treatment option reimbursable in several countries, with donislecel (Lantidra) approved specifically as a product by the US Food and Drug Administration (FDA) in 2023. Yet, deceased islet transplantation remains limited by donor organ supply and the requirement for lifelong immunosuppression. Next generation cell-based therapies, including pluripotent stem cell (PSC)-derived therapies, aim to overcome these hurdles by generating an endless supply of functional cells (Silva et al., 2022). A variety of approaches, including localized immune modulation, encapsulation, and genetic techniques, are concomitantly being pursued to overcome allogeneic immune rejection (Kioulaphides and Garcia, 2024; Rech Tondin and Lanzoni, 2025). Overall, the nonclinical regulatory expectations for efficacy and safety data (including the types and scope) for cell therapy products are relatively consistent across key regions, including US FDA, European Medicines Agency (EMA), Japan's Pharmaceuticals and Medical Devices Agency (PMDA), Australian Therapeutic Goods Administration (TGA), and Health Canada, with subtle differences in requirements between jurisdictions (see Table S1). Readers are referred elsewhere for a more comprehensive overview of relevant guidelines (Hirai et al., 2023). Most major regulators also provide regulatory and scientific advice to sponsors at various stages of the development process (see Table S2). In any case, sponsors must demonstrate that their products are effective (efficacy/pharmacology) and safe (safety/toxicology) and exhibit the expected biological properties. Nevertheless, given the complexity of these next generation product concepts, the regulatory requirements often seem hard to navigate over the full spectrum of product development from preclinical to commercialization (Beetler et al., 2023). It should be noted that both autologous and allogeneic iPSC-derived therapies require immunoprotection strategies (for allo- or auto-immune attack), with allogeneic therapies offer the advantages of "off-the-shelf" availability, lower cost, and greater manufacturing scale and consistency. Hence, we focus on allogeneic islet cell product quality and nonclinical study considerations to enable use in a clinical trial focusing on: (1) key nonclinical data needed to enable first-in-human (FIH) trials, (2) examples of recommended and alternative nonclinical testing models to characterize different islet cell therapy products, (3) process development and validation, and (4) analytical testing for characterization and quality control.

© 2026 The Authors.
Published by Elsevier Inc. on behalf of International Society for Stem Cell Research.
This is an open access article under the CC BY license (http://creativecommons.org/licenses/by/4.0/).

NONCLINICAL DATA TO SUPPORT CLINICAL TRIALS OF NOVEL ISLET CELL THERAPIES

Nonclinical safety studies for cell therapy products, particularly for locally implanted or encapsulated cell products, are inherently different from nonclinical safety programs for small molecules. In general, the scope of the nonclinical program and types of data that will be required for a novel islet cell therapy product depends on specific product attributes, such as the source material (cells/tissue, starting materials), drug product characteristics (e.g., how the cells are manipulated), combination with any non-cellular components (e.g., scaffolds or encapsulation material), excipients included in final product formulation, and route of administration. Depending on these variables, the required studies may vary but must still focus on final product efficacy and safety.

Early nonclinical studies should therefore address key scientific parameters related to the product, including its biological properties, mechanism(s) of action, potency (i.e., number of cells and/or product units needed to elicit a sufficient pharmacological effect), and efficacy (insulin production in response to stimulus). Typically, preliminary efficacy data needs to be generated with a refined product configuration before all parameters can be fully characterized. Once the clinical product is well defined, additional properties can be evaluated, including immunogenicity, toxicology, tumorigenicity, and biodistribution. This progressive approach enables critical clinical translation assessment, such as the estimation of a starting clinical dose, feasibility of the intended delivery route, justification for selected nonclinical models, and early identification of safety concerns, while allowing appropriate risk mitigation as the product advances toward clinical studies.

Source cells source considerations

Islet cell replacement therapies derived from donor pancreata have typical donor criteria considerations (adventitious agents), and these risks are managed through the Chemistry, Manufacturing, and Controls (CMC) program. Decades of safety data collected have established that islet cells isolated from donor pancreata will have very low or negligible risks associated with tumorigenicity, toxicity, and biodistribution. Therefore, the focus of the nonclinical program will be on consistency of efficacy and establishing treatment-related effects. To date, functional islet cells have been successfully derived from either embryonic or induced pluripotent stem cells (iPSCs) (Fujikura et al., 2025). iPSCs are reprogramed from somatic cells (e.g., dermal fibroblasts, peripheral blood mononuclear cells, or keratinocytes) obtained from a donor (Song et al., 2024). The reprogramming of these somatic cells into iPSCs introduces an additional stage of manufacturing—with its own critical process parameters (CPPs), quality controls, and regulatory considerations—that is not required for ESCs. For example, the International Society for Stem Cell Research (ISSCR) published standards recommend verifying the elimination of the transgene expression in newly derived human iPSC lines prior to biobanking, distribution, and experimental use (ISSCR, 2021). While a detailed discussion of GMP manufacturing for PSC-derived products is beyond the scope of this review, readers are directed to the recently updated ISSCR *Guidelines for Stem Cell Research and Clinical Translation* (2025), which provide comprehensive recommendations for the responsible development and manufacturing of PSC-based therapies (ISSCR, 2025). Additional practical guidance on bioprocessing considerations for clinical-grade iPSC generation can be found in the ISCT Emerging Regenerative Medicine Technology working group report (Song et al., 2024) and recent Quality-by-Design frameworks for PSC-derived products (Gan et al., 2025).

Therapies derived from the directed differentiation of PSCs introduce multiple new considerations compared to donor islets that should be addressed in the nonclinical safety and efficacy program. Most significant among these are the much greater risk for tumorigenicity due to genomic instability and/or the potential presence of residual PSCs in the final product.

Characterization of cells

Early phase data should focus on characterizing the cells genetically and phenotypically to confirm that they indeed exhibit the expected properties and safety profile necessary to proceed toward an islet cell product. PSC starting material should be qualified for markers of pluripotency, possibly including glycoprotein antigens SSEA-3/SSEA-4, TRA-1-60, and TRA-1-81, markers of undifferentiated human PSCs: CD9, CD90, and CD30, and transcription factors which help maintain pluripotency: OCT4, SOX2, and NANOG (Andrews et al., 1996; Henderson et al., 2002). Additional testing on source cells might include genomic (DNA) sequencing and gene expression profiling (RNA), ideally using *in vitro* cultures of the target cells. Protein expression profiling can also help ensure that the cell product exhibits the overall relevant expression profile for the starting, intermediate, or final differentiated islet cells.

For products with notable cellular heterogeneity, it may be beneficial to characterize the ratios among resulting islet cell types when starting with SC sources, such as the percentage of β, α, and off-target cell types. Additional metabolic parameters, including endocrine properties of the cells, and the presence of any contaminating cell types, should also be assessed. *In vitro* assays for whole islet

Table 1. Characteristics of biomaterials and nonclinical testing considerations

Biomaterial Strategy	Properties	Considerations for nonclinical testing
Macroencapsulation	• Capsules >1 mm in size<br>• Up to thousands of islets<br>• Hollow fiber, ultra-filtrate or planar devices<br>• Semi-permeable membrane<br>• Implanted in intravascular or extravascular space	• Increased strength of capsule<br>• Ease of access or removal<br>• Poor oxygenation<br>• Nutrient access<br>• Lag in insulin response
Micro/ nanoencapsulation	• Capsules 100 to <1000 μm in size<br>• 1-2 islets per capsule<br>• Implanted in peritoneal cavity or subcutaneous<br>• Natural matrices: agarose, alginate, collagen, chitosan<br>• Synthetic matrices: PEG, polyvinyl alcohol	• Improved transport of insulin and nutrients<br>• Improved islet viability and reduced hypoxia<br>• Poor stability of matrix<br>• Islet retrieval difficult<br>• Potential inflammatory reactions<br>• Clumping of islets<br>• Limitations on implant sites
Open scaffolds	• Highly porous materials (e.g., polycaprolactone [PCL])<br>• May enable extrahepatic transplantation<br>• Non-immunoprotective	• Improved insulin-nutrient exchange<br>• Enhanced vascularization<br>• Improved viability and oxygenation<br>• Enables retrievability<br>• Susceptible to mechanical stress<br>• No immune protection

function, including dynamic glucose-stimulated insulin secretion (GSIS), should be conducted (Li, 2020). Importantly, measures of safety, stability, cellular phenotype, and characterization also apply to co-transplanted cells such as endothelial cells, mesenchymal cells, or microvessels, if any. While these cells do not directly contribute to the mechanism of action of insulin production, they may play a supportive role in enabling the survival of the islet cells, making their inclusion and characterization key for some programs.

Finally, processes for gene editing can directly or indirectly increase the frequency of genomic sequence or copy number variants (cell stress and selection due to cloning, more population doublings). When gene editing is used as a strategy to generate immune-evasive cells, immunosuppression withdrawal (if utilized) is not expected to be a mitigation against undesired cell growth.

Encapsulation, scaffolds, and biomaterial safety

If the islet cell product is to be implanted *in vivo* using a biomaterial delivery system, such as an open scaffold or immunoisolating encapsulation, nonclinical studies must be performed to demonstrate not only islet cell survival and physiologic performance, but also biocompatibility and functional suitability of the encapsulation material. The encapsulation approach must support adequate oxygen exchange, permit free transit of insulin and other secreted factors, protect the cells from immune attack (when applicable), and maintain mechanical and structural integrity for a specified time to ensure cells remain viable and functional following implantation.

Developers of cell therapies that use biomaterial delivery systems must carefully consider the composition of the material and how it interacts with the encapsulated cells. Islet cells may be encapsulated using a variety of approaches, including immunoisolating, macro-, micro-, or nano-encapsulation, and open non-immunoprotective scaffolds (Opara et al., 2021). Each type of material comes with its own advantages and safety considerations (see Table 1) (Opara et al., 2021). Common encapsulation materials include alginate, agarose, chitosan, and collagen, and other synthetic matrices include polyethylene glycol (PEG) and polyvinyl alcohol.

Compatibility of encapsulation materials with the intended target tissues must also be evaluated *in vivo*. Unlike conventional transplantation of unencapsulated islets via intraportal hepatic infusion, biomaterial-based approaches necessitate implantation into alternative sites, such as the omentum, peritoneal cavity, subcutaneous space, or muscle (Damyar et al., 2021). As such, *in vivo* studies must assess not only how the target site supports implantation, but also how the encapsulated cells interact with the biomaterial at that site, and how the overall cell-biomaterial construct interfaces with the surrounding tissue environment. The feasibility of surgical removal of the combination product may also be demonstrated.

Taken together, combination products using biomaterials should be evaluated for (1) islet cell survival and function, (2) material integrity/degradation, (3) insulin secretion, (4) fibrotic and immune responses, (5) local irritation, and (6) extractable and leachable materials. Sponsors may refer to ICH Q3E: Guideline for Extractables and Leachables (E&L) (2020) for evaluating extractable and leachable materials (ICH, 2020).

Nonclinical efficacy studies

In vivo nonclinical efficacy studies should complement these aforementioned characterizations of the cells and encapsulation or scaffold materials. Sponsors should select models based on specific scientific questions and the biological relevance of the model, including how well the model captures key aspects of human T1D pathophysiology. This includes not only the intended pharmacological mechanism (e.g., insulin production and glucose regulation) but also host responses such as immune rejection and integration of the implanted product.

Data from nonclinical animal models should be generated to establish the mechanism of action, potency, and early safety of the cell product before initiating GLP safety studies. For the purposes of early PoC studies, the most commonly used animal models of T1D are streptozotocin (STZ)-induced models, primarily in immune-deficient rodents. Genetic models such as the autoimmune NOD mouse are valuable for studying the immune response, particularly when evaluating the autoimmune aspects of T1D but have limited utility for testing human cells due to the expected dominance of xeno-rejection mechanisms over autoimmune rejection. For more complex evaluations such as assessment of clinically relevant cell doses, delivery procedures, device performance, and host immune responses, large animal models (e.g., pigs and primates) can sometimes enhance translational relevance. These models more closely approximate human anatomy, physiology, and metabolic demand and can provide critical information to support dose selection, surgical feasibility, immune management strategies, and overall risk assessment prior to FIH studies.

Safety and biodistribution testing

Unlike small molecule safety studies, nonclinical toxicology studies for islet cell products and other cellular and gene therapy products are often single dose, but may have two or more terminal endpoints in the study (USFDA, 2013). Regulators often like to follow studies “longitudinally” over time with terminal endpoints, for example, at 1, 3, and 6 months. In practice, *in vivo* studies of 6-month duration are commonly viewed as sufficient to assess chronic safety in rodents, given their lifespan relative to humans. Study durations longer than 6–9 months are generally not anticipated, except for tumorigenicity assessments in certain cases where prolonged observation is warranted. However, the appropriate duration should be justified based on product characteristics (e.g., cell persistence and device durability) and the specific questions being addressed.

Cell doses should be based on results of nonclinical efficacy studies, and toxicology testing needs to be conducted with a dose range exceeding the proposed dose in humans based on body weight or the size of the target region of implant. Regulators will expect sponsors to provide sound justification for the proposed dose(s) and calculations presented to indicate the dose multiples compared to the proposed maximal clinical dose. In most cases for islet replacement therapies, the cell dose may be limited by the site/route of implantation, and sponsors must scale this dose to humans based on comparable body and organ size. Further, multiples of the maximal clinical dose are expected to have been evaluated cumulatively in the toxicology and tumorigenicity study(ies).

With respect to study size, regulators generally expect nonclinical group sizes sufficient to support longitudinal assessment of product safety and biological behavior. As a general benchmark, many nonclinical safety studies for cell therapy products have employed group sizes of at least 10 rodents/sex per dose level per time point, including negative controls, or 2–4 large animals per treatment group per euthanasia time point. Consistent with longitudinal study designs that emphasize serial sampling, these group sizes are intended to support assessments of general product safety over time, including monitoring toxicities, tumorigenicity, and cell biodistribution. They are not designed to detect rare adverse events but rather to characterize biological behavior and safety signals relevant to clinical translation. Cohort sizes should typically be larger than the intended final number per sex per group to account for anticipated animal attrition over the course of the study (e.g., model-specific pathologies and/or natural causes). For long-term studies, particularly in immune-deficient rodent models with known background morbidity, enrollment numbers may need to be increased substantially to ensure adequate animal numbers at later scheduled evaluations (e.g., lymphosarcoma in SCID mice for a 9-month time point). It is important to note that the examples discussed here in terms of study durations and sample sizes are for illustrative purposes, as the exact study design will depend on the individual product, model(s) chosen, and in close alignment with the regulators.

Across nonclinical efficacy and safety studies, routine in-life observations and pharmacodynamic assessments (e.g., insulin production and glucose control) should be collected longitudinally. Body weight and food consumption should be monitored in all species, while clinical

pathology (e.g., serum chemistry and hematology) should be included in large animal studies and at study termination in small rodent models. Additional immunological endpoints may be incorporated, as appropriate, to support the interpretation of host responses (e.g., cytokines, anti-donor response, etc.). Study duration should be sufficient to address both short- and long-term biological behavior of the product, considering expected cell viability, persistence, and functional activity *in vivo*.

A key toxicology endpoint for islet cell therapy is possible tumor formation, particularly when ESCs or iPSCs were used as the starting cells. Since the long-term stability of these cells is not always known, sponsors should monitor the sites of administration over time at each terminal necropsy for unusual cell differentiation, even if not necessarily tumorigenic. At study termination, implanted cells and/or devices (the entire encapsulated product when applicable) should be recovered and evaluated for viability, cell phenotype, functional performance, detailed histopathology of target and major organ systems, assessment of implantation site tissue reactions, and biodistribution of transplanted cells.

Classic pharmacokinetic studies are not generally performed for cell therapy products; instead, regulators may expect sponsors to assess the biodistribution of the product, especially if the product is administered systemically. In many cases, when the product is delivered locally and is expected to remain at the site of administration, biodistribution assessment may be waived by regulators. If biodistribution analysis is warranted, blood samples should be collected at various intervals during the study, and a panel of key tissues should be collected at study termination for each animal (at all endpoints if multiple distinct endpoints are incorporated into the study), and tissues should be frozen and processed for quantitative PCR (qPCR) or digital-droplet PCR (ddPCR). Investigators are generally advised to validate these assays down to a sensitivity of <50 copies of cell DNA per microgram (μg) of tissue genomic DNA. Sponsors can refer to ICH S12 guidance for detailed biodistribution analysis expectations (ICH, 2023b).

A tabular listing of the key endpoints to consider for safety and biodistribution studies is provided below in Table 2. For products requiring systemic immunosuppression, such as in the current islet transplantation paradigm, immunocompromised animal models are appropriate to mimic the immune-suppressed human recipient. In large animal models, however, chronic administration of systemic immunosuppressive agents (e.g., corticosteroids, tacrolimus, or sirolimus) can result in significant off-target toxicities, including increased risk of infection, malignancy, and organ-specific complications, which may confound interpretation of product-related safety findings (Campa-Carranza et al., 2022). Moreover, higher doses of traditional immunosuppressive agents are often required to prevent xenograft rejection of human cells, potentially increasing immunosuppression-related toxicities and leading to the overestimation of product-related safety risk. These considerations should be weighed against the study objectives.

Survey of key scientific topics and nonclinical models to address safety and efficacy

Given the diversity of islet cell products, sponsors may need to leverage a range of complementary *in vitro* and *in vivo* models to comprehensively assess the product, such as GSIS, cessation of secretion at low blood glucose, immunogenicity issues, safety/tolerability, and durability. Prior to initiating *in vivo* studies, sponsors should conduct a comprehensive *in vitro* assessment of the cell product, regardless of their origin, to inform model selection and study design. For cell therapy products, regulatory authorities may accept data from a single, scientifically justified animal model in support of FIH trials, provided the model is disease-relevant, and the study endpoints are meaningful and quantifiable. Use of multiple complementary models is considered best practice when feasible, with each model selected to address specific scientific or translational questions. Regardless of the model(s) used, Sponsors should demonstrate biological activity and efficacy of the islet cell product in a model that captures key aspects of the target disease. If a novel or non-standard model is proposed, sufficient data should be provided to establish its biological relevance, reproducibility, and validity for regulatory review (e.g., in an IND filing to the FDA).

When islet cell products incorporate an immune protection strategy (e.g., gene-edited islet cell therapies or immunoisolating encapsulation approaches), immunocompetent models may be necessary to demonstrate immune protection. Given the xenogeneic challenges associated with transplanting human cells into immunocompetent non-human animal models, a surrogate cell product may be developed and tested in an allogeneic model to address specific nonclinical questions. In this context, healthy, large animal species (e.g., dogs, pigs, or NHPs) may be used selectively to support the evaluation of implantation procedures, human-scale devices, and combination products. Large animal studies are typically designed as longitudinal investigations with an extended duration of follow-up, reflecting the longer life span of these species. The larger body size of these animals also permits repeated blood sampling for the assessment of insulin, glucose levels, and other clinical pathology parameters, which are often limited in rodent models. There is no requirement from regulators to conduct nonclinical safety or efficacy studies in large animal models. Decisions to include large

Table 2. Expectations and considerations for nonclinical studies of islet cell products

Type of Study	Key Objectives	Relevant Endpoints to Consider	Important Study Considerations
In vitro Experiments	• **Qualification of cell function for expected mechanism of action (e.g. insulin secretion, metabolism)**[a]<br>• **Phenotypic characterization**<br>• **Genotypic qualification and stability throughout differentiation when relevant**<br>• Characterize immune cell interactions<br>• **Qualification of cell/encapsulation biocompatibility**	• Insulin secretion<br>• Glucose utilization<br>• RNA profiling<br>• Proteomic analysis<br>• Histological analysis of islets and capsules<br>• Cell viability in 3D matrix/capsule	• Source of islets<br>• Nature of encapsulation<br>• Cell survival/compatibility with capsule<br>• Target phenotypic gene markers<br>• Off-target cell type enumeration
In vivo Efficacy	• **Rationale for animal model, route of administration and dose level**<br>• **Define active/relevant dose levels**<br>• **Translation of cell dose to humans**<br>• Establish duration and level of insulin secretion<br>• Support use of encapsulation<br>• Support for immunosuppression regimen	• Cell survival and function<br>• Insulin secretion<br>• C-peptide<br>• Glucose control<br>• Cell compatibility with material (if applicable)<br>• Immunogenicity/reaction to cells<br>• Vascularization if applicable	• Site of administration<br>• Relevance of animal model<br>• Correlation of model with human T1D disease<br>• Assays for insulin and glucose<br>• Impact on local tissues/site of implant<br>• Survival of encapsulated cells
In vivo Biodistribution	• **Characterize cell distribution and persistence**	• Selecting relevant markers or target gene sequences specificity and copy number<br>• Terminal endpoints for tissue collection<br>• Sensitivity of assay	• Nature of qPCR probe or IHC antibody for detection<br>• Study duration<br>• Number of sampling/sacrifices timepoints<br>• Tissues to collect for analysis
GLP Toxicity and Tumorigenicity	• **Product safety and tolerability**<br>• **Qualifying local and systemic toxicity Identifying dose limiting toxicity (if any)**<br>• **Assess tumorigenicity of the cells (teratoma and tumors or dysplastic cells due to residual pluripotent cells)**	• Survival<br>• Clinical signs<br>• Body weights/Food consumption<br>• Clinical pathology<br>• Gross and microscopic pathology<br>• Immune response/cytokine release<br>• Local/regional irritation<br>• Tumor formation<br>• Cell phenotypes in the graft<br>• Cell proliferation in the graft	• Species and relevance<br>• Number per group: (Rodents: 10/sex/group; Large animals 2-4/group/timepoint)<br>• Study duration, timepoints<br>• Terminal euthanasia endpoints<br>• Site/route of delivery<br>• Persistence of cells<br>• Maintenance of cell phenotype

[a]Attributes required by regulatory authorities distinguished in bold.

animals should reflect the specific scientific and translational objectives of the development program, rather than being used routinely. Table 3 provides a synthesis of key considerations and best practices when selecting nonclinical models. A more comprehensive summary of the animal models used for the study of T1D and islet cells can be found in Table 4.

For *in vivo* studies, the following guiding principles should be considered.

- When feasible and scientifically appropriate, the same transplant site and route of administration as intended for clinical testing should be used, recognizing that anatomical and physiological differences between species may necessitate adaptations.
- Efficacy studies should demonstrate the timing of functional onset, a sustained duration of product function of 4–6 months, and seek to find the time point at which the functional β cell mass achieves steady state or plateau.
- Because human-specific C-peptide and insulin ELISAs are readily available, blood glucose levels alone should never be exclusively relied upon for the demonstration of graft function.
  - o Human C-peptide and/or insulin can be used to monitor graft function in intact rodent and porcine models (not β cell-depleted).
  - o Due to its superior serum stability and lower interspecies cross-reactivity relative to insulin, C-peptide is more reproducible and preferable for evaluating graft function across different model systems and study designs, especially when blood glucose metabolism and insulin persistence may vary (Leighton et al., 2017).
- Regulatory agencies require safety testing of the Drug Product formulated per the intended clinical use. When cells are intended to be encapsulated, safety testing of the cells alone is of little value. Understandably, process evolution between early nonclinical and FIH material can be common for PSC-derived and encapsulated products. Therefore, definitive safety testing should strive to use a clinically comparable final product if strict identity is not practical.

CONSIDERATIONS FOR PROCESS DEVELOPMENT THROUGH THE VALIDATION OF T1D CELL THERAPIES

Following research process optimization, process development activities represent the initial CMC activity for the product and aim to provide clinically representative batches for nonclinical studies, including analytical development. In a situation where safety and efficacy are showing significant promise in the clinic to warrant an accelerated pathway, early validation practices can help secure regulators' agreement to permit conditional post-approval CMC commitments. This section aims to emphasize the best practices to consider during early development that can shorten future process validation timelines.

For allogenic cell products, scale-up is a significant challenge and often will be achieved in at least 2 or even 3 different scale steps (e.g., initial clinical, pivotal clinical, commercial scales). While cell culture systems have been developed to facilitate larger scales, these have mostly been developed for either 3D single cells (immune, CAR-T) or adherent cells (MSC). These systems most often need to be adapted to PSC-derived islet culture systems.

Process characterization

To effectively develop a process that is suitable for routine commercial manufacturing, process characterization (PC) activities include the standard industry practice of performing risk assessments on each stage's parameters to categorize their impact on a critical quality attribute (CQA). A process parameter is any input variable that can be directly controlled by the process. When a process parameter's variability impacts a CQA (ICH, 2009), it is considered a CPP. All CPPs being monitored or controlled for a process should be subjected to PC to deliver consistent product quality. For the development of islet cell therapies incorporating SC-derived products, the culture and differentiation process will be subject to general CPPs common to most tissue culture practices, such as temperature, oxygen concentration, and relative humidity. Table 5 provides example CQAs and CPPs for islet cell products differentiated from SCs (Balboa et al., 2022; Hogrebe et al., 2021; Pagliuca et al., 2014), and the Parental Drug Association Technical Report 42 provides additional details on the process validation of manufacturing on different constitutions of process parameters ("PDA technical report no. 42: Process validation of protein manufacturing. Parenteral Drug Association, 2005"). It is most common in the industry for biological products to have a scaled-down model representative of the full-scale model to use for PC studies.

With a cell product where the process has multiple stages (e.g., PSC-derived cell therapies), each stage's process would ideally be characterized prior to the subsequent stage's initiation of PC. For example, PSCs are thawed and expanded prior to initiating the differentiation procedure (Agulnick et al., 2015; Schulz, 2015). Establishing the process ranges for thawing the cells is important to ensure that variables, such as time and temperature, would support the expected results from the expansion even in the worst-case scenarios. Suitably validated operating ranges for thawing will allow for a consistently effective PC of

Table 3. Best practices for nonclinical safety and efficacy assessment

Study Purpose	Key Considerations	Preferred Models	Alternative Models
Toxicity, tumorigenicity, and cellular distribution	● Must permit robust survival of cells<br>● Fewest model-specific toxicities<br>● Test dose/kg higher than clinical maximum<br>● Duration at least 6 months<br>● Minimum 10 animals per sex/per group at terminal time point	● Non-diabetic immune-deficient rodent<br>● Single dose/maximal feasible dose<br>● Combine toxicity, tumorigenicity and cellular distribution in single study<br>● Rats offer benefits of higher feasible cell dose, more blood volume (all endpoints each animal)	● Large animals for surgical procedure safety, higher doses, certain transplant sites and/or large size device<br>● Requirement for systemic immunosuppression may introduce confounding toxicities and/or limit duration
Risk of excessive insulin release	● Inappropriate regulation of insulin secretion, especially at low blood glucose	● *In vitro* testing via perifusion systems<br>● *In vivo* insulin tolerance testing	● *Ex vivo* perifusion studies from explanted graft cells (Robert et al., 2018); permits evaluation of alpha cell function
Demonstrating cell engraftment and insulin production	● *In vitro* testing first, but must progress to *in vivo* demonstration<br>● Time points demonstrate functional onset, plateau and persistence at least 6 months	● Non-diabetic immune-deficient rodent (human C-peptide measurement)<br>● Basal and glucose-stimulated insulin/C-peptide secretion; may be IP injection or feeding<br>● Reduction of rodent non-fasting glucose (typically ∼110–150 mg/dL) toward the human physiological range (∼70–100 mg/dL)	● β cell-depleted immune-deficient rodents (e.g., STZ-treated)
Demonstrating cell function including physiological glucose control	● Test in the absence of endogenous β cells<br>● C-peptide/insulin measurement in addition to glucose	● Chemically induced diabetes in immune-deficient rodents<br>● STZ-treatment can be prior- or post-transplant<br>● Confirm β cell ablation by method other than hyperglycemia (e.g., histology)	● Chemical or surgical diabetes in large animal (often require systemic immunosuppression); use when rodents not suitable<br>● Surgical diabetes in large animal; requires exocrine function supplementation yet metabolic disturbances may still be substantial (e.g., malabsorption, weight loss, vitamin deficiency)
Predicting human therapeutic and sub-therapeutic doses	● Leverage extensive clinical literature for islet transplant (allo- and auto-) (Fiorina et al., 2008; Sutherland et al., 2008)<br>● Administration commonly 10,000 IEQ/kg but engrafted β cell mass maybe 10–30% of administration dose (Keymeulen et al., 2006; Korsgren et al., 2005; Ryan et al., 2001)	● *Option 1*: Resultant β cell mass by histologic or total graft lysates in fully engrafted model (e.g., immune deficient rodent)<br>● *Option 2*: Quantitative measurement of physiological function; typically requires glucose clamp (simple hyperglycemic correction does not define therapeutic dose)	● Hyperglycemic correction of a large animal similar in size to human subjects (typically porcine)

(*Continued on next page*)

Table 3. Continued

Study Purpose	Key Considerations	Preferred Models	Alternative Models
Effectiveness of immune evasion by genetic modification	● Must abrogate allo- and auto-immune rejection<br>● Typical immune-competent models not applicable to study of human cells (e.g., NOD mouse, BB rat)	● Initial testing with *in vitro* cytotoxicity assays (e.g., NK cells, T cells, ADCC)<br>● *In vivo* testing using humanized mouse models (Brehm et al., 2019; Deuse et al., 2019; Sintov et al., 2022)<br>● Sophisticated transgenic humanized mouse models enabling enhanced development of human NK cells alongside T and B cells (Aryee et al., 2022, 2023)	● Surrogate animal cell products, either primary islets or PSC-derived cells, can be used in immune competent allogenic models (e.g., NHP islet preparations (Hu et al., 2024))
Effectiveness of immune protection by encapsulation	● Eliminating cell-cell contact between graft and host immune cells protects against allograft (Kumagai-Braesch et al., 2013; Sasikala et al., 2013) and autoimmune rejection (Lee et al., 2009; Sweet et al., 2008)<br>● Demonstration of immune protection can generally be achieved with short-term *in vivo* studies (i.e., 4–8 weeks)	● Allogenic surrogate cells in immune competent species (e.g., MHC-mismatched rat islets in rats, NHP islets in NHP)<br>● Surrogate cells should approximate the human cell product in terms of oxygen requirements and regenerative potential (primary islets have failed in devices where PSC-derived therapies succeed)	● Humanized mouse models for evaluating encapsulated human cells (see above), though inherent model complexity and relative absence of robust FBR may limit clinical extrapolation (see below)
Efficacy of encapsulated products considering FBR	● Consider both FBR<br>o Fibroblast-derived capsular responses<br>o Macrophage-derived foreign body giant cell (FBGC) responses<br>● Full effect of FBR can take months to develop<br>● Studies of 4 months and ideally 6–9 months duration to demonstrate durability	● Athymic nude rats are generally an acceptable model for FBGC responses, but fibroblast/fibrous responses are usually not robust<br>● Large animals better model fibroblast/fibrous responses but device may not protect xenograft; if immunosuppression included it may attenuate the FBGC	● Specialized transgenic humanized mouse (NSG SGM3); may better model human FBGC response provided human monocyte/macrophage engraftment is robust (Doloff et al., 2023)<br>● Caution: Most mouse models have weak FBGC and fibroblast responses

Table 4. *In Vivo* animal models for efficacy evaluation of islet cell products

Animal Model	Available Species	Primary Uses	Advantages/Disadvantages of Model
Non-diabetic	Mice, rats	Efficacy studies	● Simplicity, for routine and scaled use<br>● Can easily monitor human β cell function with human-specific C-peptide/insulin ELISA<br>● Can evaluate blood glucose regulation<br>● Aligns to preferred GLP safety model
**Drug or Chemically Induced T1D Models**			
Streptozotocin model	Mice, rats, dogs, pigs, nonhuman primates	Mechanistic studies	● Well established<br>● Rapid induction of hyperglycemia, β-cell depletion without autoimmune component<br>● Used in numerous species
Alloxan model	Mice, rats	Mechanistic studies	● Destruction of islet cells *in vivo*<br>● Selective inhibition of glucose-stimulated insulin secretion<br>● Numerous species available<br>● Safety issues for researchers working with Alloxan<br>● High mortality associated with alloxan, resulting in less common use
**Genetic Models of T1D**			
NOD Mouse	Mouse	Mechanistic studies;<br>Longer-term studies compared to STZ model	● Displays hyperglycemia, immune cell infiltration and destruction of islet cells<br>● Well characterized<br>● Difficult to handle<br>● Susceptibility to infection<br>● Limited applicability to human cells (xenografts)
NSG-RIP-DTR Mouse	Mouse	Mechanistic studies	● Genetically engineered for precise, inducible, and rapid destruction of pancreatic β cells, without systemic toxicity such as STZ and Alloxan<br>● Backcrossed to the NSG background (NOD.Cg-Prkdcscid Il2rgtm1Wjl/SzJ), enabling xenotransplantation studies (Yang et al., 2015)<br>● The high sensitivity of these mice requires careful dosing of diphtheria toxin to ensure full ablation while minimizing potential non-specific side effects

(*Continued on next page*)

Table 4. *Continued*

Animal Model	Available Species	Primary Uses	Advantages/Disadvantages of Model
LETL Rat	Rat	Modeling T1D	● Spontaneous disease model<br>● Mirrors many elements of pathology and phenotype of T1D<br>● High cost<br>● Low frequency of spontaneous disease<br>● Does not mirror the progression of disease in humans<br>● Limited applicability to human cells (xenografts)
KDP Rat	Rat	Genotypic research	● Closely mirrors etiology of human T1D<br>● Rapid onset of T1D without T cell lymphoma<br>● T1D develops in both genders at 70% rate<br>● Expensive to maintain/set up<br>● Limited applicability to human cells (xenografts)
Large Animals	Dog, Pig, Nonhuman Primate	Surgical feasibility, implantation strategy, device performance and supportive efficacy assessment	● Comparable anatomy/physiology to humans<br>● Enable systems-level evaluation or glucose-insulin dynamics, metabolic demand, and host responses<br>● Allow implantation of human-scale devices and clinically relevant cell doses<br>● Amenable to longitudinal sampling and functional assessments<br>● Handling challenges<br>● Use is program-specific and not for regulatory approval<br>● High cost and operational complexity

Table 5. Example CQA and CPP for a generalized multistage stem cell-derived islet differentiation protocol

Stage Number	Stage	Example CQA	Example CPP
0	Pluripotent state	OCT4, NANOG positivity	• Seeding density<br>• Time in culture<br>• Base media composition
1	Definitive endoderm	>90% SOX17, FOXA2 positivity	• Activin A and CHIR concentration<br>• Stage length (hours, days)
2	Primitive gut tube	–	• KGF concentration<br>• Stage length (hours, days)
3	Posterior foregut / Pancreatic progenitor	>80% PDX1 positivity<br>Morphology	• Retinoic acid concentration<br>• Stage length (hours, days)
4	Pancreatic progenitor	>40% PDX1, NKX6.1 positivity	• Retinoic acid concentration<br>• Stage length (hours, days)
5	Endocrine progenitor	>80% CHGA positivity	• Gamma-secretase inhibitor, thyroid hormone, and EGF concentrations<br>• Stage length (hours, days)
6	Pancreatic islet	>20% NKX6.1, C-peptide positivity<br>Dithizone staining<br>Morphology	• [a] Cell dissociation time and temperature<br>• [a] Cell cluster formation efficiency, size distribution<br>• Cell density ([a] of aggregates/cells per media volume)<br>• Stage length (hours, days)

[a]CPP associated with cell cluster formation may apply to any stage where 3D aggregates are formed

the cell expansion. Each unit of operation in the expansion process should be interrogated to define suitable parameters that effectively capture an operating range that produces an expanded cell product ready for the subsequent aggregation or differentiation step.

This approach of completing characterization for the input materials stage by stage, which includes the cells from the previous stage as well as the reagents used for the current stage, minimizes the risk of needing to return more than a single stage due to unforeseen issues during PC of the subsequent stage. When a stage is having difficulty establishing controlled conditions to support its characterization, it is likely that a key attribute from the immediate previous step, or less likely from earlier steps, was not appropriately accounted for. In turn, this would warrant consideration for additional in-process controls (IPCs) and characterization of the step around this attribute to help control or mitigate any impact from uncharacterized conditions.

Having a representative scaled-down model is ideal for any PC campaign. Usually, the transition from a scaled-down model to the full-scale model marks the end of PC. But in some instances, only the full-scale model can accommodate certain range-finding study criteria. To help define which scale to use during a product's campaign toward validation, best practices include performing a gap analysis following the lockdown of the process description as defined by the subject matter experts and allowed by the process design space (see Figure S1).

In-process controls (IPCs)

Considering PSC-derived cell products have a greater propensity for cell type heterogeneity in the final product (relative to donor-derived cell therapies), the analytics between the differentiation stages provide valuable, if not essential, occasions to define CQAs that further support the constitution of the final cell product. As such, it is central to the process to establish assays early during development that enable informed decision-making practices through each of the process's differentiation stages. While IPC point testing will not commonly be used as a product release specification, this testing is nonetheless critical and will serve as the backbone for PC and validation studies.

Process-related impurities

The goal is to produce a cell product with maximum efficacy while accounting for process-related residuals to mitigate and minimize any potential toxicity due to residuals. All regulatory bodies will consider residuals in the final product during the safety profile assessment. The potential impact of various types of process residuals is summarized in Table S3.

Final product profile: Gene expression, function, and product residuals

When a therapeutic is starting from a pluripotent cell source, the manufacturing process invariably transitions through multiple intermediate cell types and therefore the gene expression profiles are highly dynamic throughout manufacturing. These differences are key to understanding each stage's units of operations that will be required to establish control over the process and ultimately for achieving a validated process. Practices to adopt early include deep analytical characterization of the cellular starting material to help elucidate genotypic and phenotypic attributes that distinguish it from the downstream products (Kroon et al., 2008; Nair et al., 2020).

Table S3 provides a representation of general categories for product-related impurities (e.g., non-target cell types) in islet cell products and methods for the measurement of impurities associated with undifferentiated cells, non-target intermediate cell types, and dead cells, but testing for product-related impurities may not be limited to these categories. When it comes to undifferentiated cells, testing should include at least one marker identifying pluripotent cells (Dobner et al., 2024), and it may be beneficial to include a marker that evaluates cell proliferation, such as Ki-67 or PCNA. Genetic stability needs to be tested and typically includes final product cells as well as earlier cell banks. In cases where other supporting cell types (e.g., endothelial cells, non-β endocrine cells) in addition to β cells are also expected in the final cell product, purity testing should be performed for every expected target cell type. This expectation for final cell product-related impurity testing is consistent across multiple regulatory bodies (EMA, 2015; 2018; PMDA, 2025; Simon et al., 2024; USFDA, 2024a; 2024c), and discussions with the regulatory agencies are an opportunity to obtain feedback on which product-related impurity testing method(s) are most suitable for lot release or which can be maintained for characterization.

CONSIDERATIONS FOR QUALITY CONTROL AND ANALYTICAL TESTING OF T1D CELL THERAPIES

Analytical testing for T1D cell replacement therapies is required to demonstrate the safety, identity, purity, and quality of the product. In addition to release testing of the drug product, testing of the cell banks, raw materials, and intermediates, including applicable drug substances, is a critical part of ensuring overall product quality. The required testing is based on product type, route of administration, and product knowledge. Testing requirements can vary based on the phase of drug development and generally become more stringent as a product moves further in development. This roadmap focuses primarily on quality control and analytical considerations for early drug development, including definitive preclinical studies to Phase 1 clinical trials.

Cell banks

Following the reprogramming of allogeneic iPSCs, it is critical to note that clone selection is an important early decision that could impact many downstream parameters. A balance between several critical factors is required for the final clone selection, which may include genetic integrity, growth behavior, and islet function as key parameters to assess. Once clone selection is completed, Master and Working Cell Banks (MCB, WCB) must be created and tested to ensure the quality and suitability of the substrate for use in WCB or drug product manufacturing, respectively. Testing will vary based on the specific biology of the cells, but generally includes testing for identity and purity, adventitious or endogenous agents, and molecular contaminants, such as residual reprogramming components in the case of iPSCs. Genetically modified cell lines should be tested to ensure the presence of gene modification(s) and potentially consistency of expression for transgenes, if appropriate. Examples of testing for MCBs and WCBs with sample acceptance criteria are shown in Table S4. Additional considerations for the testing of cell banks are discussed in ICH Q5D (ICH, 1998).

Starting materials and raw materials

Cellular materials are considered starting materials when used for further drug product manufacturing. For allogeneic donor material, manufacturers are required to determine and document whether a donor is eligible based upon the results of donor screening and donor testing, each being a different component of eligibility. General requirements include the collection of the test specimen up to 7 days before or after the collection of donor material. In the case of donors of peripheral blood stem/progenitor cells or bone marrow, the specimen for testing may be collected up to 30 days before the collection of donor material. Notably, Japan's PMDA does not mandate a fixed time range from collection to use for donor testing. Rather, it requires risk-based screening and repeat testing around "window periods," which are defined as the early infection stages during which pathogens (viruses, bacteria, etc.) may not yet be detectable by tests (PMDA, 2024).

"Raw material" is a general term that encompasses different classifications of materials used for the manufacture of drug substances and drug products. It is imperative to have an early understanding of the differences between ancillary raw materials and excipients, as the regulatory testing and reporting requirements differ for each type of raw material (Table S5).

Ancillary raw materials should be monitored for clearance throughout the manufacturing process, as they are considered process-related impurities if present in the final drug substance and/or drug product.

For all drug products, including early phase products, excipients must be GMP or USP grade materials. However, there is greater flexibility in the materials that are intended for use as ancillary materials. It is not uncommon that early-phase drug products require the use of ancillary materials that are not provided as a GMP or USP grade and transitioned to GMP grade materials for late stage and commercial manufacturing. To enhance product safety when using research-grade materials, control strategies for these materials should include.

- Instituting a supplier qualification program and performing supplier audits
- Confirming COA test results that are critical to product safety with testing upon receipt
- Verification that animal- or human-derived materials are free of adventitious agents
- Performance testing for residual materials in the final drug substance or drug product
- Maintaining a risk assessment log (per ICH Q9) to justify material selections and alternatives (ICH, 2021).

For additional guidance on a risk-based approach in qualifying ancillary materials, see USP <1043> Ancillary Materials for Cell, Gene, and Tissue-Engineered Products (USP, 2019).

Compatibility between cells and materials or encapsulation devices

The requirements for biocompatibility and stability of device materials are generally well established according to medical device regulations and described by ISO 10993 (ISO, 2018). These include the impact of the materials on the host and, to a lesser extent, the impact of the host on the materials. For cell-device combination products, whether encapsulation or open scaffolds, testing should also be performed to establish the compatibility of the cell therapy with the materials with which they are combined. In its most basic form, this will entail viability testing for the cells after formulation with the device materials as the final drug product. Depending on the nature of the materials and their potential to be bioactive, additional testing of cell phenotype and function may also be required.

It should be noted that the definition of a combination product is different between the EU and the US. Combined ATMP is defined (integral device part of the active substance) by EMA, but there is no legal definition of a combination product, in contrast to the US, where the definition is wide-ranging (USFDA, 2018). When the device component is not integral, the label will indicate "a medicine used with a medical device" (e.g., non-integral or referenced). A medicine with an integral device is classified as a medicine, which impacts data requirements, but not the classification.

Under the EU legislation, encapsulation of cells constitutes a manufacturing step, which would render the product an ATMP. It is advised that the developer should seek a classification meeting request with the EMA's Committee for Advanced Therapies (EMA, 2025a).

Analytical methods and testing

In general, all safety and quality requirements must be met for all phases of product development, including general characteristics, purity, identity, potency, and safety. Where there may be flexibility is in the final versions of certain analytical assays, such as potency where a surrogate test may be used in the early phase while the product is being developed and proven, prior to investing in often more complex and expensive assays (e.g., cell-based bioassays). It is also imperative to consider orthogonal assays where appropriate.

The FDA approval of donislecel provides insights regarding release and stability testing expectations for final drug products (USFDA, 2023b). For example, the use of a visual islet morphology test for identity was approved, and the use of flow cytometry was suggested as a consideration but not required. Such a precedent may be leveraged if it is also scientifically supported and validated for the product under development. In this example, the use of a visual identity test may be acceptable for next-generation T1D cell replacement products, but it is advised to implement flow cytometry as soon as possible, as this has become an emerging best practice for the analytical assessment of cell therapies. Similar arguments can be made for purity and viability assessments. For example, although hemocytometry is the standard for initial IND approval by the U.S. FDA, automated cell counting and viability assessments, although not in any established regulatory guidance, are increasingly utilized and could be expected by regulatory authorities, as they become aligned with ICH Q2(R2) and Q14 (ICH, 2023a; 2023c).

Similar consideration should be given to drug substance (s) as well as drug product lot release and stability testing. Next-generation products may include one or more drug substances, such as purified cellular material, nanofibers, and/or hydrogels, prior to the encapsulation process. The fully encapsulated material would therefore be considered the drug product, whereas the individual components may be treated as drug substances from an analytical and regulatory perspective. The individual drug substances would also be subject to full release and stability testing prior to encapsulation. Table 6 provides lot release and

Table 6. Lot release and stability testing considerations for T1D cell therapy drug products

	T1D Cell Therapy Drug Products		
**Quality Attribute**	**Allogeneic islet cells from donor pancreas**	**Allogeneic stem cell-derived islet cells**	**Encapsulated allogeneic stem cell-derived pancreatic progenitors**
Appearance	Visual appearance for color, clarity, and particulates	Visual appearance for color, clarity, and particulates	Visual appearance for color, clarity, and particulates
Cell Count & Viability	Total cell count reported; cell viability $\geq$70%	Automated cell count; viability $\geq$70%	Automated cell count and viability of cells, before and after encapsulation
Identity	Visual islet morphology	Flow cytometry marker(s) for identity of islet cells	Flow cytometry marker(s) for identity of pancreatic cells, before and after encapsulation
Purity	Islet purity (Lantidra uses visual microscopy at $\geq$30%)(USFDA, 2023b)	Flow cytometry marker(s) for islet cell purity<br>% islet cells and % common impurities present (e.g., residual PSC)	Flow cytometry marker(s) for pancreatic progenitor cell purity<br>% pancreatic progenitor & endocrine cells and % common impurities present (e.g., residual PSC), before and after encapsulation
Potency	Glucose static incubation (Insulin secretion quantitated by ELISA)	Glucose static incubation (Insulin secretion quantitated by ELISA) or similar activity assay based on mechanism of action	Glucose static incubation (Insulin secretion quantitated by ELISA) or similar activity assay based on mechanism of action, before and after encapsulation
Safety	Sterility by USP<71>	Sterility by USP<71>	Sterility by USP<71>
	Gram stain[a]	Gram stain[a]	Gram stain[a]
	Bacterial endotoxins by USP<85>	Bacterial endotoxins by USP<85>	Bacterial endotoxins by USP<85>
Additional tests depending on delivery	N/A	N/A	Microencapsulation (e.g., polymers, hydrogels)<br>Device/scaffold will have additional testing required depending on encapsulation materials used[b]

(*Continued on next page*)

Table 6. Continued

	T1D Cell Therapy Drug Products		
**Quality Attribute**	**Allogeneic islet cells from donor pancreas**	**Allogeneic stem cell-derived islet cells**	**Encapsulated allogeneic stem cell-derived pancreatic progenitors**
Characterization tests	FDA recommendations include:<br>● Retrospective evaluation of ductal cells and non-β cells in final DP<br>● Flow cytometry<br>● HLA analysis to be considered	Potential expression of stem cell-derived islet markers, such as:<br>● PDX1<br>● NKX6-1<br>● CHGA<br>● INS<br>● SIX2<br>● MAFB<br>Could use PCR, ELISA or flow cytometry as appropriate.<br>Potential expression of non-islet cell types<br>Consider mature cell type distribution, such as mature vs. immature endocrine progenitors[c]<br>Absence of acquired genomic variants throughout differentiation process	Same characterization as stem cell-derived islet cells, plus additional characterization of encapsulation materials as needed and appropriate

[a]Gram stain testing is performed as a rapid assessment of sterility for release prior to the availability of the 14-day USP<71> sterility test.
[b]Devices are regulated under sections 201(g) and 201(h) of the FD&C Act (21 USC 321(g) and (h) Scaffolds manufactured using biomaterials such as natural and synthetic polymers (e.g., collagen, hyaluronic acid, and polyethylene glycol) will also need to be tested for their identity and stability; test method(s) will depend on the specific biomaterials used.
[c]Some of these characterization tests may also need to be validated for QC identity and purity testing (see EMA) (ICH, 2023c).

stability testing considerations for drug products. Islet drug products can serve as a standalone product comparator, and depending on the PSC-derived product type/stage, primary islets may also serve as an appropriate analytical benchmark, provided the significant lot-to-lot variability of islet preparations is accounted for.

It is worth noting that all methods must be fit for purpose at all phases of development. The determination of fit for purpose will entail either the verification, qualification, or validation of each lot release and stability test method. Verification of test methods entails the determination of the suitability of using compendial methods, such as bacterial endotoxins and sterility.

Qualification and validation generally refer to non-compendial test methods, which are test methods that are product-specific, such as purity, identity, and potency. While the definitions of qualification and validation of analytical test methods can vary, here, validation is defined as the protocol-driven determination that is performed for Phase 3 and commercial readiness.

Qualification of analytical methods is typically a smaller version of validation; often, method qualifications entail a protocol-driven examination of all method parameters (e.g., precision, accuracy, and specificity) with the exception of robustness. Notably, all methods that generate data to support lot disposition and dosing (e.g., cell counts) must, at a minimum, be qualified prior to IND submission. The IND submission should include the method qualification parameters examined, along with the pre-approved criteria and qualification results.

Where feasible, developers are generally advised to establish an appropriately characterized in-house primary reference material to evaluate the performance of an analytical method and to ensure the reliability of the result obtained. The use of assay-specific control or assay-specific reference material instead of reference material, prepared from lot(s) representative of production and clinical materials, is acceptable where justified.

Characterization testing differs from release and stability testing in that characterization assays are not required for lot release of the drug substance or drug product. Characterization testing is used for several purposes. First, data from characterization testing are used to better understand the product throughout development. Product understanding and knowledge are imperative throughout all phases of development. Second, characterization testing is used to explore methods that may eventually become release and/or stability tests. Third, assays may be implemented for the purposes of characterization of the drug product for the establishment of reference materials and for comparability assessments as development progresses. Examples of characterization testing for the drug product that may be implemented in early development include PCR- or flow cytometry-based biomarker analysis for cellular differentiation (Table 5). In short, characterization testing should be used to better understand and define the drug product.

Potency

Potency testing is required for all phases of development, including the initial IND submission. The potency assay, which is often the most challenging to develop and institute, should be considered as early as possible during preclinical development. While the potency assay(s) may change over time and throughout product development, a qualified potency assay is required for Phase 1. A surrogate potency measure may be implemented for phase 1, such as an ELISA- or PCR-based method. However, it is expected that the development of a cell-based bioassay(s) that mimics the biological mechanism of action should be developed in later phases of product development. More commonly, multi-parameter potency assays are often developed as a “matrix,” which assesses identity, composition, as well as functional readouts with correlation to specific CQAs. With the end goal in mind, the exploration and development of multiple potency assays should continue throughout the entire product life cycle, starting with the preclinical development stage.

Multiple potency tests may be required for lot release and stability testing, depending on the product; as such, the determination of product potency may be required to be evaluated using a matrix of assays. The specific cellular and gene therapy product requirements and expectations for phase 1 INDs are outlined in the recent FDA draft guidance (USFDA, 2023a).

For current cellular products in T1D that comprise glucose-responsive β cells, the GSIS assay is the gold standard. Insulin secretion assays should continue to be used for allogeneic islet cells or SC-derived islet cell drug products. The assessment of potency for encapsulated drug products may be more challenging. Cellular drug substance should be assessed using GSIS assays, while an encapsulated drug product may require a more complex cell-based bioassay approach, such as testing of encapsulated and non-encapsulated islet cells from the same batch in parallel.

Genetic stability and genetic variants

The application of PSCs for scaled and reproducible production of replacement islets is promising, in part due to their extensive proliferative potential, but the magnitude of necessary population doublings inherent to these cell sources will generate genetic variants. Certain aspects of PSC biology contribute to the acquisition of genetic variants, including chromosome mitotic segregation errors, a short G1 cell cycle phase, and hypertranscriptional states (Vales and Barbaric, 2024).

In accordance with the reported propensity of genomic changes and the availability of the tools for detecting these changes, regulatory agencies are actively evolving in perspectives and guidance for the use and even requirement for deep genomic interrogation of PSC-derived cell therapies. While the understanding of the functional consequences for some of the genetic aberrations observed in PSCs is beginning to be unraveled (Andrews et al., 2022), it lacks the ability to detect genetic variants. Therefore, sponsors should evaluate the application of genetic testing to their product and discuss with regulatory agencies.

The best practice for genetic variant detection will involve the use of technologies to permit the elucidation of both sequence and copy number variation, with consideration given to the ability to detect mosaicism within the population. Traditional cytogenetic evaluation is expected to be part of cell bank analysis (USFDA, 1998). Further, consideration should be given to the points during the cell line derivation, cell banking, and cell product manufacture at which the genome is interrogated leveraging the understanding of process points where the majority of population doublings may occur (e.g., expanding from a single cell clone) and/or where culture selection pressures may be present (e.g., process points where extensive cell death has occurred). It should also be noted that the application of gene editing introduces additional manipulations that can directly (off-target cutting) or indirectly (extensive time in culture, cloning, etc.) increase the frequency of acquired genetic variants, including abnormal ploidy.

Encapsulation and combination products

Drug products for T1D cell-based therapies, where the cells are not the final product, relegate the cells to a drug substance status (e.g., encapsulated products). Demonstration of potency conferring attributes for an encapsulated product can be challenging, and getting agency feedback on testing strategies early in development is recommended. If a biomaterial is not considered a device, the manufacturing of the biomaterial will need to have its process characterized appropriately to provide a biomaterial suitable for the PC of the final drug product.

The current regulatory environment for classifying a drug as a combination product is evolving. Advances in technology and material sciences often bring novel combination approaches that warrant alternative classifications or exemptions from typical regulatory frameworks. For cell-based drugs uniquely combined with biomaterials, developers are encouraged to engage early with regulatory agencies for product education and to help assure appropriate designation with respect to combination product status.

CONCLUDING REMARKS

Nonclinical development of pancreatic islet cell therapies is a complex process, heavily dependent on the nature and design of the individual product. To help operationalize the concepts discussed throughout this review, Table S6 provides some illustrative examples from product attributes to key nonclinical questions, preferred models, potential safety risks, potency strategies, and CMC/QC priorities. The key to a successful nonclinical program is selecting the most relevant *in vitro* and *in vivo* models. GLP-compliant safety studies should be carefully designed with input from regulators and performed in a well-controlled setting. Early efficacy studies should be sufficiently rigorous to inform important development decisions. A well-designed and executed nonclinical program will reduce product development risks and thus reduce the overall time-to-clinic.

A successful development program also demands a proactive, strategic, and phase-appropriate CMC approach. Exemplary process and analytical strategies can reduce the initial time-to-clinic, such as by significantly reducing unintended studies (e.g., comparability), while also paving the way for a commercially viable product. Thus, careful attention to process development and analytical testing is essential for success. By integrating quality-by-design principles, establishing robust control strategies early, and engaging with regulatory agencies, sponsors can build a solid foundation that significantly accelerates these novel, life-changing islet cell therapies to patients.

ACKNOWLEDGMENTS

We thank Breakthrough T1D staff member Brianna Greeno for supporting the document review and quality control, and Ellen G. Feigal, NDA Partners, LLC, for critical reviews of the manuscript. Individual studies were supported by funding as reported previously in references cited for each study. Breakthrough T1D funded and supported the development and writing of the manuscript.

AUTHOR CONTRIBUTIONS

Conceptualization, M.M. and E.L.; methodology, M.M., C.P., and K.D.; writing – K.D., C.P., N.M., D.M.C., D.P., and R.H.K.; writing – review and editing, K.D., C.P., N.M., D.M.C., D.P., R.H.K., N.H., E. L., M.L.G., M.A.B., and M.M.; supervision, M.M.

DECLARATION OF INTERESTS

Esther Latres is a former employee of Regeneron Pharmaceuticals and owns company stock. Melanie L. Graham receives funding from the NIH (USA; grant numbers R01AR078624, U19AI174966, R42AR083779, R21AI90721, U01AI126322, R21AI182508), Breakthrough T1D (2-SRA-2025-1648-S-B), and Regenerative Medicine Minnesota (RMM, 202501, RMM, 20240311TR033). Kevin D'Amour, David Pepperl, Robert H. Kutner, and Diana M. Colleluori

received paid consulting fees from Breakthrough T1D for contributing to this work. No other potential conflicts of interest relevant to this article were reported.

SUPPLEMENTAL INFORMATION

Supplemental information can be found online at https://doi.org/10.1016/j.stemcr.2026.102998.

REFERENCES

Agulnick, A.D., Ambruzs, D.M., Moorman, M.A., Bhoumik, A., Cesario, R.M., Payne, J.K., Kelly, J.R., Haakmeester, C., Srijemac, R., Wilson, A.Z., et al. (2015). Insulin-Producing Endocrine Cells Differentiated In Vitro From Human Embryonic Stem Cells Function in Macroencapsulation Devices In Vivo. Stem Cells Transl. Med. *4*, 1214–1222. https://doi.org/10.5966/sctm.2015-0079.

Andrews, P.W., Barbaric, I., Benvenisty, N., Draper, J.S., Ludwig, T., Merkle, F.T., Sato, Y., Spits, C., Stacey, G.N., Wang, H., and Pera, M. F. (2022). The consequences of recurrent genetic and epigenetic variants in human pluripotent stem cells. Cell Stem Cell *29*, 1624–1636. https://doi.org/10.1016/j.stem.2022.11.006.

Andrews, P.W., Casper, J., Damjanov, I., Duggan-Keen, M., Giwercman, A., Hata, J.i., von Keitz, A., Looijenga, L.H.J., Millan, J.L., Oosterhuis, J.W., et al. (1996). Comparative analysis of cell surface antigens expressed by cell lines derived from human germ cell tumours. Int. J. Cancer *66*, 806–816. https://doi.org/10.1002/(SICI)1097-0215(19960611)66:6<806::AID-IJC17>3.0.CO;2-0.

Aryee, K.E., Burzenski, L.M., Yao, L.C., Keck, J.G., Greiner, D.L., Shultz, L.D., and Brehm, M.A. (2022). Enhanced development of functional human NK cells in NOD-scid-IL2rg(null) mice expressing human IL15. FASEB J. *36*, e22476. https://doi.org/10.1096/fj.202200045R.

Aryee, K.E., Shultz, L.D., Burzenski, L.M., Greiner, D.L., and Brehm, M.A. (2023). NOD-scid IL2rgammanull mice lacking TLR4 support human immune system development and the study of human-specific innate immunity. J. Leukoc. Biol. *113*, 418–433. https://doi.org/10.1093/jleuko/qiac020.

Balboa, D., Barsby, T., Lithovius, V., Saarimäki-Vire, J., Omar-Hmeadi, M., Dyachok, O., Montaser, H., Lund, P.E., Yang, M., Ibrahim, H., et al. (2022). Functional, metabolic and transcriptional maturation of human pancreatic islets derived from stem cells. Nat. Biotechnol. *40*, 1042–1055. https://doi.org/10.1038/s41587-022-01219-z.

Beetler, D.J., Di Florio, D.N., Law, E.W., Groen, C.M., Windebank, A.J., Peterson, Q.P., and Fairweather, D. (2023). The evolving regulatory landscape in regenerative medicine. Mol. Aspect. Med. *91*, 101138. https://doi.org/10.1016/j.mam.2022.101138.

Brehm, M.A., Kenney, L.L., Wiles, M.V., Low, B.E., Tisch, R.M., Burzenski, L., Mueller, C., Greiner, D.L., and Shultz, L.D. (2019). Lack of acute xenogeneic graft- versus-host disease, but retention of T-cell function following engraftment of human peripheral blood mononuclear cells in NSG mice deficient in MHC class I and II expression. FASEB J. *33*, 3137–3151. https://doi.org/10.1096/fj.201800636R.

Bortell, R., Yang, C., Loehn, M., Jurczyk, A., Przewozniak, N., Leehy, L., Herrera, P., Shultz, L., Greiner, D., and Harlan, D. (2015). Lixisenatide accelerates restoration of normoglycemia and improves human beta-cell function and survival in diabetic immunodeficient NOD-scid IL-2rg(null) RIP-DTR mice engrafted with human islets. Diabetes Metab. Syndr. Obes. *8*, 387–398. https://doi.org/10.2147/DMSO.S87253.

Campa-Carranza, J.N., Paez-Mayorga, J., Chua, C.Y.X., Nichols, J. E., and Grattoni, A. (2022). Emerging local immunomodulatory strategies to circumvent systemic immunosuppression in cell transplantation. Expert Opin. Drug Deliv. *19*, 595–610. https://doi.org/10.1080/17425247.2022.2076834.

Damyar, K., Farahmand, V., Whaley, D., Alexander, M., and Lakey, J.R.T. (2021). An overview of current advancements in pancreatic islet transplantation into the omentum. Islets *13*, 115–120. https://doi.org/10.1080/19382014.2021.1954459.

Deuse, T., Hu, X., Gravina, A., Wang, D., Tediashvili, G., De, C., Thayer, W.O., Wahl, A., Garcia, J.V., Reichenspurner, H., et al. (2019). Hypoimmunogenic derivatives of induced pluripotent stem cells evade immune rejection in fully immunocompetent allogeneic recipients. Nat. Biotechnol. *37*, 252–258. https://doi.org/10.1038/s41587-019-0016-3.

Dobner, J., Diecke, S., Krutmann, J., Prigione, A., and Rossi, A. (2024). Reassessment of marker genes in human induced pluripotent stem cells for enhanced quality control. Nat. Commun. *15*, 8547. https://doi.org/10.1038/s41467-024-52922-1.

Doloff, J.C., Ma, M., Sadraei, A., Tam, H.H., Farah, S., Hollister-Lock, J., Vegas, A.J., Veiseh, O., Quiroz, V.M., Rakoski, A., et al. (2023). Identification of a humanized mouse model for functional testing of immune-mediated biomaterial foreign body response. Sci. Adv. *9*, eade9488. https://doi.org/10.1126/sciadv.ade9488.

EMA (2015). Reflection paper on classification of advanced therapy medicinal products. https://www.ema.europa.eu/en/documents/scientific-guideline/reflection-paper-classification-advanced-therapy-medicinal-products_en.pdf-0.

EMA (2018). Guideline on the quality, non-clinical and clinical aspects of gene therapy medicinal products. https://www.ema.europa.eu/en/documents/scientific-guideline/guideline-quality-non-clinical-and-clinical-aspects-gene-therapy-medicinal-products_en.pdf.

EMA (2025a). Advanced therapy medicinal products: Overview. https://www.ema.europa.eu/en/human-regulatory-overview/advanced-therapy-medicinal-products-overview#:∼:text=Stem%20cells%20are%20naturally%20occurring,approval%20in%20the%20European%20Union.

Fiorina, P., Shapiro, A.M.J., Ricordi, C., and Secchi, A. (2008). The clinical impact of islet transplantation. Am. J. Transplant. *8*, 1990–1997. https://doi.org/10.1111/j.1600-6143.2008.02353.x.

Fujikura, J., Anazawa, T., Toyoda, T., Ito, R., Kimura, Y., and Yabe, D. (2025). Toward a cure for diabetes: iPSC and ESC-derived islet cell transplantation trials. J. Diabetes Investig. *16*, 384–388. https://doi.org/10.1111/jdi.14366.

Galloway, J., and Chance, R. (1994). Improving insulin therapy: achievements and challenges. Horm. Metab. Res. *26*, 591–598. https://doi.org/10.1055/s-2007-1001766.

Gan, Y., Wang, Y., Wang, S., and Zhang, Y.A. (2025). A Quality By Design Approach of Developing a Human Pluripotent Stem Cell Derived Cell Therapy Product. Adv. Exp. Med. Biol. *1486*, 305–319. https://doi.org/10.1007/978-3-031-97297-3_23.

Grattoni, A., Korbutt, G., Tomei, A.A., García, A.J., Pepper, A.R., Stabler, C., Brehm, M., Papas, K., Citro, A., Shirwan, H., et al. (2025). Harnessing cellular therapeutics for type 1 diabetes mellitus: progress, challenges, and the road ahead. Nat. Rev. Endocrinol. *21*, 14–30. https://doi.org/10.1038/s41574-024-01029-0.

Henderson, J.K., Draper, J.S., Baillie, H.S., Fishel, S., Thomson, J.A., Moore, H., and Andrews, P.W. (2002). Preimplantation human embryos and embryonic stem cells show comparable expression of stage-specific embryonic antigens. Stem Cell. *20*, 329–337. https://doi.org/10.1634/stemcells.20-4-329.

Hirai, T., Yasuda, S., Umezawa, A., and Sato, Y. (2023). Country-specific regulation and international standardization of cell-based therapeutic products derived from pluripotent stem cells. Stem Cell Rep. *18*, 1573–1591. https://doi.org/10.1016/j.stemcr.2023.05.003.

Hogrebe, N.J., Maxwell, K.G., Augsornworawat, P., and Millman, J. R. (2021). Generation of insulin-producing pancreatic beta cells from multiple human stem cell lines. Nat. Protoc. *16*, 4109–4143. https://doi.org/10.1038/s41596-021-00560-y.

Hu, X., White, K., Olroyd, A.G., DeJesus, R., Dominguez, A.A., Dowdle, W.E., Friera, A.M., Young, C., Wells, F., Chu, E.Y., et al. (2024). Hypoimmune induced pluripotent stem cells survive long term in fully immunocompetent, allogeneic rhesus macaques. Nat. Biotechnol. *42*, 413–423. https://doi.org/10.1038/s41587-023-01784-x.

ICH (1998). ICH Q5D Derivation and characterisation of cell substrates used for production of biotechnological/biological products - Scientific guideline. https://www.ema.europa.eu/en/ich-q5d-derivation-characterisation-cell-substrates-used-production-biotechnological-biological-products-scientific-guideline.

ICH (2009). ICH HARMONISED TRIPARTITE GUIDELINE PHARMACEUTICAL DEVELOPMENT Q8(R2). https://database.ich.org/sites/default/files/Q8_R2_Guideline.pdf.

ICH (2020). GUIDELINE FOR EXTRACTABLES AND LEACHABLES Q3E. https://database.ich.org/sites/default/files/ICH_Q3E_EWG_Step2_DraftGuideline_2025_0704.pdf.

ICH (2021). ICH HARMONISED GUIDELINE: QUALITY RISK MANAGEMENT Q9(R1). https://database.ich.org/sites/default/files/ICH_Q9-R1_Document_Step2_Guideline_2021_1118.pdf.

ICH (2023a). ICH HARMONISED GUIDELINE: ANALYTICAL PROCEDURE DEVELOPMENT Q14. https://database.ich.org/sites/default/files/ICH_Q14_Guideline_2023_1116.pdf.

ICH (2023b). ICH HARMONISED GUIDELINE: NONCLINICAL BIODISTRIBUTION CONSIDERATIONS FOR GENE THERAPY PRODUCTS S12. https://database.ich.org/sites/default/files/ICH_S12_Step4_Guideline_2023_0314.pdf.

ICH (2023c). ICH Q2(R2) Validation of analytical procedures - Scientific guideline. https://www.ema.europa.eu/en/ich-q2r2-validation-analytical-procedures-scientific-guideline.

ISO (2018). Biological evaluation of medical devices Part 1: Evaluation and testing within a risk management process. https://www.iso.org/standard/68936.html.

ISSCR (2021). Basic Research Standards. In Section 1: Basic Characterization. https://www.isscr.org/basic-research-standards/basic-characterization#:~:text=Some%20methods%20for%20authenticating%20cell%20lines%20include:,of%20registries%20include:%20*%20**Cellosaurus**%20*%20**hPSCreg**:ISSCR.

ISSCR (2025). Guidelines for Stem Cell Research and Clinical Translation. In Version 1.2. https://www.isscr.org/guidelines:ISSCR.

Kerper, N., Ashe, S., and Hebrok, M. (2022). Pancreatic beta-Cell Development and Regeneration. Cold Spring Harbor Perspect. Biol. *14*, a040741. https://doi.org/10.1101/cshperspect.a040741.

Keymeulen, B., Gillard, P., Mathieu, C., Movahedi, B., Maleux, G., Delvaux, G., Ysebaert, D., Roep, B., Vandemeulebroucke, E., Marichal, M., et al. (2006). Correlation between beta cell mass and glycemic control in type 1 diabetic recipients of islet cell graft. Proc. Natl. Acad. Sci. USA *103*, 17444–17449. https://doi.org/10.1073/pnas.0608141103.

Kioulaphides, S., and García, A.J. (2024). Encapsulation and immune protection for type 1 diabetes cell therapy. Adv. Drug Deliv. Rev. *207*, 115205. https://doi.org/10.1016/j.addr.2024.115205.

Korsgren, O., Nilsson, B., Berne, C., Felldin, M., Foss, A., Kallen, R., Lundgren, T., Salmela, K., Tibell, A., and Tufveson, G. (2005). Current status of clinical islet transplantation. Transplantation *79*, 1289–1293. https://doi.org/10.1097/01.tp.0000157273.60147.7c.

Kroon, E., Martinson, L.A., Kadoya, K., Bang, A.G., Kelly, O.G., Eliazer, S., Young, H., Richardson, M., Smart, N.G., Cunningham, J., et al. (2008). Pancreatic endoderm derived from human embryonic stem cells generates glucose-responsive insulin-secreting cells in vivo. Nat. Biotechnol. *26*, 443–452. https://doi.org/10.1038/nbt1393.

Kumagai-Braesch, M., Jacobson, S., Mori, H., Jia, X., Takahashi, T., Wernerson, A., Flodström-Tullberg, M., and Tibell, A. (2013). The TheraCyte device protects against islet allograft rejection in immunized hosts. Cell Transplant. *22*, 1137–1146. https://doi.org/10.3727/096368912X657486.

Lee, S.H., Hao, E., Savinov, A.Y., Geron, I., Strongin, A.Y., and Itkin-Ansari, P. (2009). Human beta-cell precursors mature into functional insulin-producing cells in an immunoisolation device: implications for diabetes cell therapies. Transplantation *87*, 983–991. https://doi.org/10.1097/TP.0b013e31819c86ea.

Leighton, E., Sainsbury, C.A., and Jones, G.C. (2017). A Practical Review of C-Peptide Testing in Diabetes. Diabetes Ther. *8*, 475–487. https://doi.org/10.1007/s13300-017-0265-4.

Li, W.H. (2020). Functional analysis of islet cells in vitro, in situ, and in vivo. Semin. Cell Dev. Biol. *103*, 14–19. https://doi.org/10.1016/j.semcdb.2020.02.002.

Nair, G.G., Tzanakakis, E.S., and Hebrok, M. (2020). Emerging routes to the generation of functional beta-cells for diabetes mellitus cell therapy. Nat. Rev. Endocrinol. *16*, 506–518. https://doi.org/10.1038/s41574-020-0375-3.

Nathan, D.M., and Group, D.E.R. (2014). The diabetes control and complications trial/epidemiology of diabetes interventions and

complications study at 30 years: overview. Diabetes Care *37*, 9–16. https://doi.org/10.2337/dc13-2112.

Opara, A., Jost, A., Dagogo-Jack, S., and Opara, E.C. (2021). Islet cell encapsulation - Application in diabetes treatment. Exp. Biol. Med. *246*, 2570–2578. https://doi.org/10.1177/15353702211040503.

Pagliuca, F.W., Millman, J.R., Gürtler, M., Segel, M., Van Dervort, A., Ryu, J.H., Peterson, Q.P., Greiner, D., and Melton, D.A. (2014). Generation of functional human pancreatic beta cells in vitro. Cell *159*, 428–439. https://doi.org/10.1016/j.cell.2014.09.040.

(2005). PDA technical report no. 42: Process validation of protein manufacturing. Parenteral drug Association. PDA J. Pharm. Sci. Technol. 59, 1–28.

PMDA (2024). STANDARDS FOR BIOLOGICAL RAW MATERIALS. https://www.pmda.go.jp/files/000268475.pdf. https://www.pmda.go.jp/files/000268475.pdf.

PMDA (2025). Regenerative Medical Products. https://www.pmda.go.jp/english/review-services/reviews/0003.html.

Rech Tondin, A., and Lanzoni, G. (2025). Islet Cell Replacement and Regeneration for Type 1 Diabetes: Current Developments and Future Prospects. BioDrugs *39*, 261–280. https://doi.org/10.1007/s40259-025-00703-7.

Robert, T., De Mesmaeker, I., Stangé, G.M., Suenens, K.G., Ling, Z., Kroon, E.J., and Pipeleers, D.G. (2018). Functional Beta Cell Mass from Device-Encapsulated hESC-Derived Pancreatic Endoderm Achieving Metabolic Control. Stem Cell Rep. *10*, 739–750. https://doi.org/10.1016/j.stemcr.2018.01.040.

Ryan, E.A., Lakey, J.R.T., Rajotte, R.V., Korbutt, G.S., Kin, T., Imes, S., Rabinovitch, A., Elliott, J.F., Bigam, D., Kneteman, N.M., et al. (2001). Clinical outcomes and insulin secretion after islet transplantation with the Edmonton protocol. Diabetes *50*, 710–719. https://doi.org/10.2337/diabetes.50.4.710.

Sasikala, M., Rao, G.V., Vijayalakshmi, V., Pradeep, R., Pothani, S., Kumar, P.P., Gaddipati, R., Sirisha, G., Cheemalakonda, R., Tandan, M., et al. (2013). Long-term functions of encapsulated islets grafted in nonhuman primates without immunosuppression. Transplantation *96*, 624–632. https://doi.org/10.1097/TP.0b013e31829e26cf.

Schulz, T.C. (2015). Concise Review: Manufacturing of Pancreatic Endoderm Cells for Clinical Trials in Type 1 Diabetes. Stem Cells Transl. Med. *4*, 927–931. https://doi.org/10.5966/sctm.2015-0058.

Silva, I.B.B., Kimura, C.H., Colantoni, V.P., and Sogayar, M.C. (2022). Stem cells differentiation into insulin-producing cells (IPCs): recent advances and current challenges. Stem Cell Res. Ther. *13*, 309. https://doi.org/10.1186/s13287-022-02977-y.

Simon, C.G., Jr., Bozenhardt, E.H., Celluzzi, C.M., Dobnik, D., Grant, M.L., Lakshmipathy, U., Nebel, T., Peltier, L., Ratcliffe, A., Sherley, J.L., et al. (2024). Mechanism of action, potency and efficacy: considerations for cell therapies. J. Transl. Med. *22*, 416. https://doi.org/10.1186/s12967-024-05179-7.

Sintov, E., Nikolskiy, I., Barrera, V., Hyoje-Ryu Kenty, J., Atkin, A.S., Gerace, D., Ho Sui, S.J., Boulanger, K., and Melton, D.A. (2022). Whole-genome CRISPR screening identifies genetic manipulations to reduce immune rejection of stem cell-derived islets. Stem Cell Rep. *17*, 1976–1990. https://doi.org/10.1016/j.stemcr.2022.08.002.

Song, H.W., Solomon, J.N., Masri, F., Ph D, Mack, A., Durand, N., Cameau, E., Dianat, N., Hunter, A., Oh, S., Schoen, B., et al. (2024). Bioprocessing considerations for generation of iPSCs intended for clinical application: perspectives from the ISCT Emerging Regenerative Medicine Technology working group. Cytotherapy *26*, 1275–1284. https://doi.org/10.1016/j.jcyt.2024.05.024.

Sutherland, D.E.R., Gruessner, A.C., Carlson, A.M., Blondet, J.J., Balamurugan, A.N., Reigstad, K.F., Beilman, G.J., Bellin, M.D., and Hering, B.J. (2008). Islet autotransplant outcomes after total pancreatectomy: a contrast to islet allograft outcomes. Transplantation *86*, 1799–1802. https://doi.org/10.1097/TP.0b013e31819143ec.

Sweet, I.R., Yanay, O., Waldron, L., Gilbert, M., Fuller, J.M., Tupling, T., Lernmark, A., and Osborne, W.R.A. (2008). Treatment of diabetic rats with encapsulated islets. J. Cell Mol. Med. *12*, 2644–2650. https://doi.org/10.1111/j.1582-4934.2008.00322.x.

USFDA (1998). Guidance for Industry: Guidance for Human Somatic Cell Therapy and Gene Therapy. https://www.fda.gov/regulatory-information/search-fda-guidance-documents/guidance-human-somatic-cell-therapy-and-gene-therapy.

USFDA (2013). Guidance for Industry: Preclinical Assessment of Investigational Cellular and Gene Therapy Products. https://www.fda.gov/regulatory-information/search-fda-guidance-documents/preclinical-assessment-investigational-cellular-and-gene-therapy-products.

USFDA (2018). Combination Product Definition Combination Product Types. https://www.fda.gov/combination-products/about-combination-products/combination-product-definition-combination-product-types.

USFDA (2023a). GUIDANCE DOCUMENT: Potency Assurance for Cellular and Gene Therapy Products. https://www.fda.gov/regulatory-information/search-fda-guidance-documents/potency-assurance-cellular-and-gene-therapy-products.

USFDA (2023b). LANTIDRA STN: 125734. https://www.fda.gov/vaccines-blood-biologics/lantidra.

USFDA (2024a). GUIDANCE DOCUMENT: Considerations for the Use of Human-and Animal-Derived Materials in the Manufacture of Cell and Gene Therapy and Tissue-Engineered Medical Products. https://www.fda.gov/regulatory-information/search-fda-guidance-documents/considerations-use-human-and-animal-derived-materials-manufacture-cell-and-gene-therapy-and-tissue.

USFDA (2024c). GUIDANCE DOCUMENT: Safety Testing of Human Allogeneic Cells Expanded for Use in Cell-Based Medical Products. https://www.fda.gov/regulatory-information/search-fda-guidance-documents/safety-testing-human-allogeneic-cells-expanded-use-cell-based-medical-products.

USP (2019). Ancillary Materials for Cell, Gene, and Tissue-Engineered Products. https://doi.usp.org/USPNF/USPNF_M620_02_01.html.

Vales, J.P., and Barbaric, I. (2024). Culture-acquired genetic variation in human pluripotent stem cells: Twenty years on. Bioessays *46*, e2400062. https://doi.org/10.1002/bies.202400062.

Stem Cell Reports, Volume *21*

Supplemental Information

Allogeneic islet products for type 1 diabetes: Navigating nonclinical and manufacturing regulatory expectations

Chengyuan Press, Kevin D'Amour, Nicholas Mamrak, David Pepperl, Robert H. Kutner, Diana M. Colleluori, Melanie L. Graham, Michael A. Brehm, Nasir Hussain, Esther Latres, and Marjana Marinac

Figure S1: Example Flow Chart for Product Process Validation

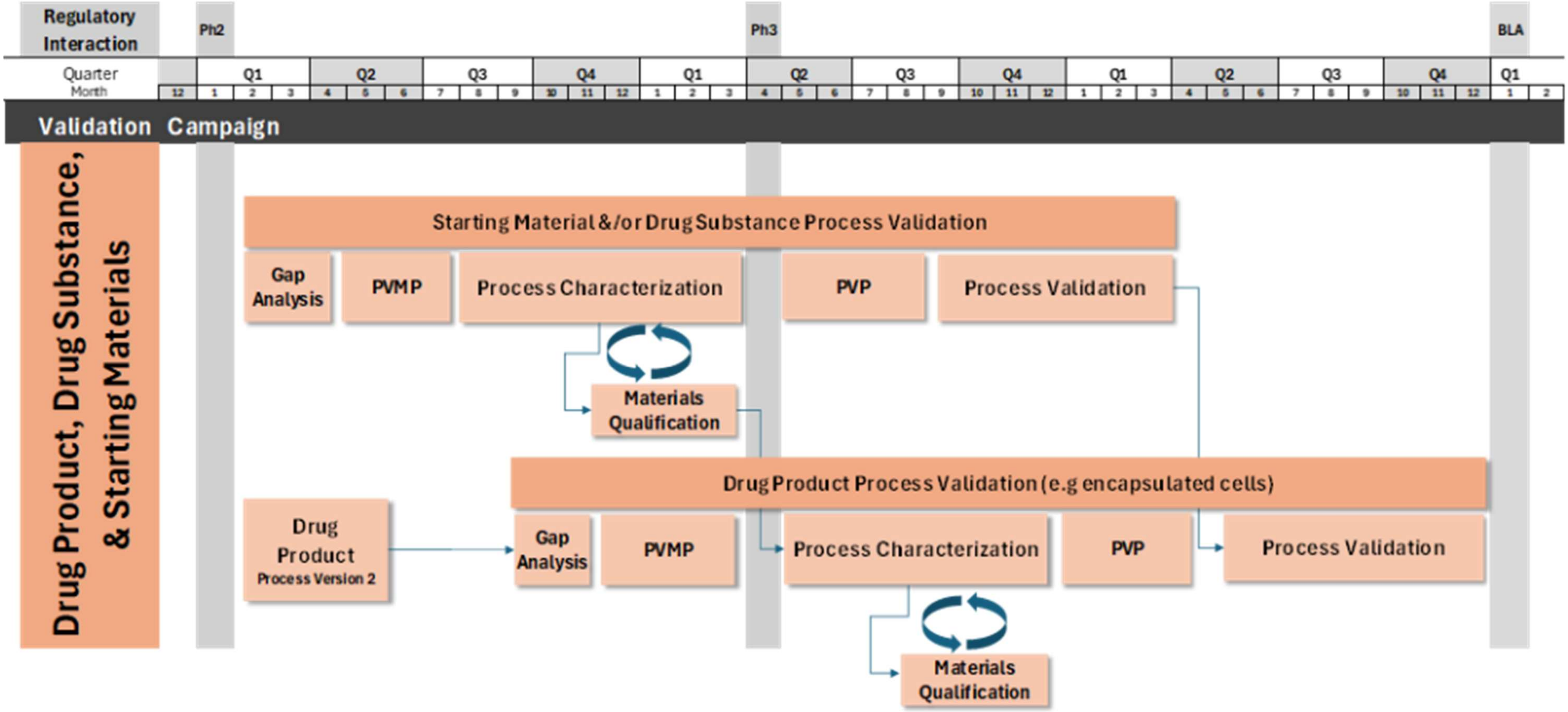

PVP: Process Validation Protocol; PVMP: Process Validation Master Protocol.

Table S1:Nonclinical Regulatory Guidelines for Cellular Therapy Products Across Worldwide Jurisdictions

Region	Regulatory Authority	Product Classification(s)	Regulatory Filing for Clinical Trial	Relevant Regional Guidance**
United States	FDA	CTs<br>GTs<br>TEPs	IND (Investigational New Drug Application) (USFDA, 1995)	• Preclinical Assessment of Investigational Cellular and Gene Therapy Products (USFDA, 2013)<br>o Outlines comprehensive preclinical assessment, focusing on product characterization, mechanism of action, proof-of-concept, biodistribution, toxicology, and dosage for clinical trials<br>• Frequently Asked Questions — Developing Potential Cellular and Gene Therapy Products (USFDA, 2024)<br>o Provides targeted answers for developing CGT products; Considerations for interacting with the FDA (including IND submissions), CMC, nonclinical studies (animal models/toxicology), and clinical trial design/safety
European Union	EMA	ATMP:<br>• GTMPs<br>• SCTMPs<br>• TEMPs	CTA (Clinical Trial Application)	• Guideline on quality, nonclinical and clinical requirements for investigational advanced therapy medicinal products in clinical trials (2024) (EMA, 2025)<br>o Quality control, safety studies, minimally required nonclinical data, and clinical trial design via risk-based approaches for early and late-phase trials
Japan	PMDA	RPs	CTA (Clinical Trial Application)	• Technical Guidance for Quality, Nonclinical Safety Studies and Clinical Studies of Regenerative Medical Products (Human Cell-Processed Products) (2016) (PMDA, 2016)<br>o Essential standards for quality control, nonclinical safety assessment (including tumorigenicity and biodistribution), and clinical study design for human cell-processed products
Australia	TGA	Biologicals:<br>• Class 1<br>• Class 2<br>• Class 3<br>• Class 4	CTA (Clinical Trial Application) or CTN (Clinical Trial Notification scheme)	• Australian Regulatory Guidelines for Biologicals (ARGB) (2018) (TGA, 2018)<br>o Risk-based classification system, manufacturing requirements, and market authorisation processes
Canada	Health Canada	ATPs	CTA (Clinical Trial Application)	• Safety of Human Cells, Tissues and Organs for Transplantation Regulations (2007) (HealthCanada, 2025)<br>o Establish mandatory safety standards for the processing, storage, distribution, and importation of human cells, tissues, and organs (CTO) to prevent disease transmission<br>o Donor suitability assessment (screening/testing), quality management (personnel, facilities), mandatory registration with Health Canada, and error/adverse reaction reporting

FDA: Food and Drug Administration; EMA: European Medicines Agency; PMDA: Pharmaceutical and Medical Devices Agency; CT: Cell Therapy; GT: Gene Therapy; TEP: Tissue Engineered Product; ATMPs: Advanced Therapy Medicinal Product; GTMP: Gene Therapy Medicinal Product; SCTMP: Somatic cell therapy medicinal product; TEMP: tissue engineered medicinal product; RP: Regenerative Medicine products; ATPs: Advanced Therapeutic Products. TGA: Therapeutic Goods Administration. *Typically, in addition to general safety and biodistribution. * and **are not exhaustive lists but the authors' highlights.

Table S2:Comparison of Scientific Meetings and Submissions with Worldwide Regulatory Authorities

**Jurisdiction**	**Early Regulatory Input**	**Accelerated Pathways and Designations**	**Key Features**
USA (FDA)	INTERACT (very early)<br>Pre-IND (prior to IND)	RMAT (Regenerative Medicine)<br>Breakthrough Therapy<br>Fast Track<br>Accelerated Approval<br>Priority Review	• INTERACT meetings for very early-stage products with challenges<br>• Pre-IND meetings help ensure adequacy of IND filing<br>• No fees or costs to any early-stage FDA meetings.<br>• Numerous opportunities for obtaining input and accelerating development process
EU (EMA)	Scientific Advice (SA) Meeting	PRIME (similar to FDA RMAT)<br>Conditional Marketing Authorization<br>Approval under Exceptional Use Marketing Authorization	• Scientific Advice from individual Nations or from Full EU<br>• National scientific advice can vary between jurisdictions, fees may be required<br>• SA from EU takes longer, but more comprehensive, associated with fees<br>• Additional IMPD filing contains comprehensive CMC/nonclinical data<br>• PRIME comparable to FDA's RMAT for regenerative medicine products
Japan (PMDA)	Pre-submission Consult	SAKIGAKE (like FDA Breakthrough Therapy)	• CTA review comparable to US FDA (30 days)<br>• SAKIGAKE pathways affords additional access to PMDA
Australia (TGA)	Pre-submission Meeting	Priority Review<br>Provisional Approval	• Novel, high-risk, FIH therapies such as gene-edited or encapsulated stem cell-derived islets would require CTA unless the FIH trial has been approved by a comparable regulator, then the CTN pathway may be used<br>• CTA involves TGA review of nonclinical and CMC data<br>• GMP compliance for FIH trials is exempted but is required beyond FIH trials, applicable to both CTN and CTA pathways
Canada (Health Canada)	Pre-CTA	Priority Review	• CTA Process highly similar to US IND filing<br>• Priority review speeds review of marketing application<br>• No fees or costs to any early-stage FDA meetings

IMPD: Investigational Medicinal Product Dossier; INTERACT: **I**nitial **T**argeted **E**ngagement for **R**egulatory **A**dvice on **C**BER Produc**t**s

Table S3: Potential Impact of Process Residuals

Cell Product and Process-Related Impurities		
**Process-related impurity**	**Method for Measurement**	**Potential Toxicity**
Feeder cells	qPCR using amplicons specific to feeder cell genome sequences	Immunogenicity
Recombinant or animal-derived proteins	ELISA ideally specific to protein	Immunogenicity
Small molecule reagents	HPLC or GC-MS	Direct toxicity
Synthetic mRNA/pDNA	qRT/PCR using amplicons specific for starting material sequences	Genotoxicity, Immunogenicity
Transfection reagents	HPLC differentiating peak-area response times	Direct toxicity
Sorting or purification reagents (e.g., antibodies or beads)	Imaging or secondary sorting measures	Immunogenicity
**Product-related impurities**		
Undifferentiated or incompletely differentiated cells	Flow cytometry for proliferation markers and/or other cell specific markers	Tumorigenicity
Dead cells	Trypan-blue exclusion	Immunogenicity

Table S4: Sample acceptance criteria for MCB and WCB Testing

Test	Acceptance Criteria	
	**Master Cell Bank**	**Working Cell Bank**
Identity	Conforms to specific cell species and donor<br><br>STR profiling	Conforms to specific cell species and donor<br><br>STR profile matches MCB
Cell Growth	Report %viability and total cells	Report %viability and total cells
Potency	Confirmed ability to form cell type of interest	Confirmed ability to form cell type of interest
Genetic Stability	Normal karyotyping<br><br>Molecular testing performed without variants of concern (e.g., NGS or SNP array)	Karyotyping consistent with MCB banding<br><br>Consistent with MCB results
Sterility	No growth	No growth
Mycoplasma	None detected	None detected
Adventitious Agents (*in vivo*)	No adventitious agents detected	N/A
Adventitious Agents (*in vitro*)	No adventitious agents detected	No adventitious agents detected
Viral Contaminants	Free of viral contaminants	N/A
Reprogramming Contaminants*	Clearance of plasmid or viral components (<1 copy per 100 cells)	N/A

STR: Short Tandem Repeat. N/A: Not Applicable, as tests are not generally required for the WCB; *: iPSC-specific consideration (Sullivan et al., 2018)

Table S5: Material Qualification and Testing Requirements

Category	Example(s)	Incoming QC Testing Requirements[1]	Material Qualification Requirements[2]	Re-Qualification[3]
GMP Critical	Primary container/closure	Identify / Appearance<br>Dimension	Full CoA testing of 3 lots, e.g., dimensions, material construction	Annual – Full CoA testing of 1 lot
	Compendial, final formulation, excipient, transport	Appearance	Full CoA testing of 3 lots	Annual – Full CoA testing of 1 lot
	Non-compendial final formulation, transport	Every lot – Full CoA testing	Full CoA testing of 3 lots	Full CoA testing was performed on each incoming receipt
	Primary printed labels	Confirm readability, lot number, expiry accuracy, artwork	Full CoA testing of 3 lots, e.g., dimensions, adhesion, label integrity / functional rub, artwork	Annual – Full CoA testing of 1 lot
Ancillary Tier 1	Licensed product	Appearance, identity	N/A	N/A
Ancillary Tier 2	Compendial grade	Appearance, identity	Full CoA testing or 3 lots	Annual – Full CoA testing of 1 lot
Ancillary Tier 3	Animal-human derived (where the supplier does perform all applicable virus and/or donor level testing)	Appearance, identity	Full CoA testing or 3 lots	Annual – Full CoA testing of 1 lot
Ancillary Tier 4	Animal-human derived (where the supplier does not perform all applicable virus and/or donor-level testing)	Appearance, identity	Full CoA testing or 3 lots and applicable virus testing per 9CFR113 and/or donor-level testing per regulations	Annual – Full CoA testing of 1 lot and applicable virus testing per 9CFR113 and/or donor-level testing per regulations
Single-Use Disposable	Scalpel, Tubing, Filters	N/A	N/A	N/A
Laboratory Reagents	Media, serum	N/A	N/A	N/A

1. For Phase 1 and 2 only verifying the CoA by a GMP-compliant vendor against Raw Materials Specification is typically acceptable for all categories.
2. For Phase 1 and 2 only the Ancillary Tier 4 requirements are expected to meet compliance, all other categories are N/A. It is recommended that such approach is also supported by an internal safety risk assessment.
3. Required for Phase 3 and beyond.

Table S6: Illustrative Examples for Practical Implementation: Mapping Product Attributes to Development Priorities

Product Attributes	Key Nonclinical Question(s)	Preferred Model(s)	Potential Safety Concern	Potency Strategy	CMC/QC Priority
PSC-Derived β-Cells (unprotected)	• Engraftment*<br>• Glucose-responsive function<br>• Dose*<br>• Off-target cell persistence*	• STZ-immune deficient rodent (cell function, long-term, 6+ months)<br>• Non-diabetic immune-deficient rodent (tumorigenicity/distribution)	• Residual PSCs → teratoma*<br>• Genomic instability*	• GSIS*<br>• Markers (e.g., INS, GCG)*	• Residual PSCs and undifferentiated cells (flow cytometry, qPCR)*<br>• Genetic stability (karyotyping, CNV analysis)*
PSC-Derived Progenitors (e.g., pancreatic endoderm)	• *In vivo* maturation timeline<br>• Fate mapping<br>• Ectopic differentiation	• STZ-immune deficient rodent (cell function, long-term, 6+ months)<br>• Non-diabetic immune-deficient rodent (tumorigenicity/distribution)	• Uncontrolled proliferation<br>• Off-target differentiation	• Functional potency after maturation<br>• Markers (e.g., PDX1+, NKX6.1+)	• *In vitro* differentiation to functional endocrine cells<br>• Markers/correlates of *in vivo* maturation potential
Gene-Edited Immune Evasion (e.g., HLA knockout)	• Protection from allo- & autoimmunity<br>• Off-target editing consequences	• Humanized mouse model (e.g., NSG with human immune system)<br>• *In vitro* cytotoxicity assays	• Off-target genomic edits<br>• Insertional mutagenesis<br>• Unanticipated immunogenicity	• Immune evasion (e.g., resistance to alloreactive T cell killing *in vitro*)	• Editing fidelity (targeted or WGS)<br>• Karyotypic stability post-editing<br>• Off-target analysis (e.g., GUIDE-seq)
Encapsulated (immunoisolating)	• Device integrity<br>• FBR<br>• Insulin diffusion kinetics<br>• Route of administration/implant site	• Allogeneic surrogate in immune-competent animal for FBR (e.g., rat, pig)<br>• Immune-deficient rodent for xenograft function	• Fibrotic overgrowth → graft failure<br>• Device rupture<br>• Leachables	• GSIS (pre- and post-encapsulation)<br>• Device integrity<br>• Diffusion (*in vitro* release assay)	• Encapsulation material characterization (biocompatibility ISO 10993)<br>• E&L (ICH Q3E); sterility

*Applies to all categories listed under "Product Attributes" in the table.

References:

EMA. (2025). *Guideline on quality, non-clinical and clinical requirements for investigational advanced therapy medicinal products in clinical trials.* https://www.ema.europa.eu/en/documents/scientific-guideline/guideline-quality-non-clinical-clinical-requirements-investigational-advanced-therapy-medicinal-products-clinical-trials_en.pdf Retrieved from https://www.ema.europa.eu/en/documents/scientific-guideline/guideline-quality-non-clinical-clinical-requirements-investigational-advanced-therapy-medicinal-products-clinical-trials_en.pdf

HealthCanada. (2025). *Safety of Human Cells, Tissues and Organs for Transplantation Regulations (SOR/2007-118).* Retrieved December 3 from https://laws-lois.justice.gc.ca/eng/regulations/sor-2007-118/index.html#:~:text=Table%20of%20Contents,65%20%2D%20Facilities

PMDA. (2016). *Technical Guidance for Quality, Nonclinical Safety Studies and Clinical Studies of Regenerative Medical Products (Human Cell-Processed Products)*. https://www.pmda.go.jp/files/000273883.pdf Retrieved from https://www.pmda.go.jp/files/000273883.pdf

Sullivan, S., Stacey, G. N., Akazawa, C., Aoyama, N., Baptista, R., Bedford, P., Bennaceur Griscelli, A., Chandra, A., Elwood, N., Girard, M., Kawamata, S., Hanatani, T., Latsis, T., Lin, S., Ludwig, T. E., Malygina, T., Mack, A., Mountford, J. C., Noggle, S.,…Song, J. (2018). Quality control guidelines for clinical-grade human induced pluripotent stem cell lines. *Regen Med*, *13*(7), 859–866. https://doi.org/10.2217/rme-2018-0095

TGA. (2018). *Classifying biologicals*. Retrieved December 3 from https://www.tga.gov.au/resources/guidance/classifying-biologicals

USFDA. (1995). *Guidance for Industry: Content and Format of Investigational New Drug Applications (INDs) for Phase 1 Studies of Drugs, Including Well-Characterized, Therapeutic, Biotechnology-derived Products*. https://www.fda.gov/regulatory-information/search-fda-guidance-documents/content-and-format-investigational-new-drug-applications-inds-phase-1-studies-drugs-including-well Retrieved from https://www.fda.gov/regulatory-information/search-fda-guidance-documents/content-and-format-investigational-new-drug-applications-inds-phase-1-studies-drugs-including-well

USFDA. (2013). *Guidance for Industry: Preclinical Assessment of Investigational Cellular and Gene Therapy Products.* https://www.fda.gov/regulatory-information/search-fda-guidance-documents/preclinical-assessment-investigational-cellular-and-gene-therapy-products Retrieved from https://www.fda.gov/regulatory-information/search-fda-guidance-documents/preclinical-assessment-investigational-cellular-and-gene-therapy-products

USFDA. (2024). *GUIDANCE DOCUMENT: Frequently Asked Questions — Developing Potential Cellular and Gene Therapy Products.* https://www.fda.gov/regulatory-information/search-fda-guidance-documents/frequently-asked-questions-developing-potential-cellular-and-gene-therapy-products Retrieved from https://www.fda.gov/regulatory-information/search-fda-guidance-documents/frequently-asked-questions-developing-potential-cellular-and-gene-therapy-products
